# Effect of Treatment with Interferon Beta-1a on Changes in Voxel-Wise Magnetization Transfer Ratio in Normal Appearing Brain Tissue and Lesions of Patients with Relapsing–Remitting Multiple Sclerosis: A 24-Week, Controlled Pilot Study

**DOI:** 10.1371/journal.pone.0091098

**Published:** 2014-03-13

**Authors:** Robert Zivadinov, Michael G. Dwyer, Silva Markovic-Plese, Cheryl Kennedy, Niels Bergsland, Deepa P. Ramasamy, Jacqueline Durfee, David Hojnacki, Brooke Hayward, Fernando Dangond, Bianca Weinstock-Guttman

**Affiliations:** 1 Buffalo Neuroimaging Analysis Center, Department of Neurology, State University of New York at Buffalo, Buffalo, New York, United States of America; 2 Department of Neurology, State University of New York at Buffalo, Buffalo, New York, United States of America; 3 Department of Neurology, Microbiology and Immunology, University of North Carolina at Chapel Hill, Chapel Hill, North Carolina, United States of America; 4 EMD Serono, Inc., Rockland, Massachusetts, United States of America; University of Oxford, United Kingdom

## Abstract

**Background:**

This pilot study investigated changes in remyelinating and demyelinating activity in normal appearing brain tissue (NABT) and lesions, by using voxel-wise magnetization transfer ratio (VW-MTR), in patients with relapsing–remitting multiple sclerosis (RRMS) receiving interferon beta-1a 44 mcg subcutaneously (IFN β-1a SC) three times weekly versus healthy controls (HCs) (NCT01085318).

**Methods:**

Increasing (suggestive of remyelination) and decreasing (suggestive of demyelination) VW-MTR changes in NABT and in T2, T1 and gadolinium (Gd)-enhancing lesion volume were measured over 24 weeks in 23 patients treated with IFN β-1a SC and in 15 HCs (where applicable). VW-MTR changes were tested using the Wilcoxon signed–rank or Wilcoxon rank–sum test.

**Results:**

A trend for greater volume of NABT with increasing VW-MTR at 24 weeks was observed for patients versus HCs (median [range] 1206 [0–15278]; 342 [0–951] mm^3^; p = 0.061). NABT volume with increasing VW-MTR at 12 weeks was significantly greater in patients than in HCs (852 [6–11577]; 360 [0–1755] mm^3^; p = 0.028). Similar findings were detected for lesion volumes. Two patients with notably high numbers of Gd-enhancing lesions at baseline had a markedly greater volume of tissue with increasing VW-MTR compared with other patients. Volume of NABT tissue with decreasing VW-MTR was significantly greater in patients versus HCs at 24 weeks (942 [0–6141]; 297 [0–852] mm^3^; p<0.001).

**Conclusions:**

The significant change in NABT volume with increasing VW-MTR at 12 weeks suggests that active remyelination in patients with RRMS may occur during treatment with IFN β-1a SC. Findings from two patients with the highest number of Gd-enhancing lesions at baseline suggest that extensive remyelination in NABT may occur in patients with high disease activity. Tissue volume with decreasing VW-MTR was greater in patients than in HCs, despite treatment, validating the sensitivity of this technique for detecting MS disease activity.

**Trial Registration:**

ClinicalTrials.gov NCT01085318.

## Introduction

The hallmarks of multiple sclerosis (MS) are inflammation and neurodegeneration, with the ongoing processes of demyelination and remyelination occurring both focally, in central nervous system lesions, and diffusely, in normal appearing brain tissue (NABT) [1]–[3]. Magnetization transfer ratio (MTR) analysis of myelin content allows quantitative detection of processes in the brain suggestive of demyelination and remyelination *in vivo*, by measuring and quantifying the degree of signal loss at each voxel [4]–[6]. However, conventional MTR analysis relies on *a priori* location assumptions, and is therefore insensitive to changes that might occur outside of the predefined boundaries. Importantly, the technique is sensitive only to mean change in the analyzed area rather than for the overall level of activity; this creates a scenario where in many *a priori* regions of interest (ROI), competing processes of demyelination and remyelination may cancel each other out, resulting in a measurement falsely suggesting a lack of disease activity [7], [8].

In contrast, voxel-wise MTR (VW-MTR) analysis is an advanced image processing technique that measures each voxel independently and assesses direction and degree of change, thus avoiding the need for defining *a priori* ROI. This process also increases the chance to detect competing processes of demyelination and remyelination *in vivo*, allowing a more in-depth understanding of the dynamism of MS disease processes [4], [7]–[11]. In addition, tracking VW-MTR changes may provide a means for integrating information about a variety of tissue changes not individually detectable with any other single magnetic resonance imaging (MRI) modality [7], [8].

Remyelination usually occurs, to a variable extent, over several months following lesion formation [10]. Early stages of remyelination were observed in patients with acute or early MS, whereas remyelination in later progressive MS was thought to be sparse, with varying degrees of remyelination competing with demyelination in MS lesions. In secondary-progressive MS, more demyelination and higher brain lesion volume (LV) of active demyelination were observed, compared with patients with primary-progressive MS [12]. Goldschmidt et al. also reported significantly more remyelinated lesions in biopsies of patients with early MS compared with autopsy cases of chronic MS [13]. Conversely, histopathological analysis of autopsied, completely and partially remyelinated lesions, conducted by Patrikios et al., showed that remyelination was not restricted to early stages of the disease and did occur in progressive MS [14].

In murine models, myelin loss, macrophage infiltration, axonal damage and increase in water content are manifested as decreasing MTR, whereas increasing MTR may result from remyelination, although potential decreases in water content may also play a role [5], [15]–[17]. However, in six patients with relapsing–remitting MS (RRMS) and acute gadolinium (Gd)-enhancing lesions, while the edema in the acute lesions attenuated the decrease in MTR, a strong correlation between the changes in MTR and myelin content remained [18]. Several studies describe the extent of increases and decreases of VW-MTR signal within NABT as suggestive of remyelinating and demyelinating processes, respectively, in MS following treatment with immunomodulatory therapy [7], [8], [10], [16]. Pivotal and subsequent clinical trials showed that interferon beta-1a (IFN β-1a) given subcutaneously (SC) three times weekly (tiw) at 22 and 44 mcg in patients with RRMS, decreased inflammatory MRI activity and clinical disease activity, as measured by reduction of relapses and slowing in progression of disability [19], [20]. However, the effect of IFN β-1a SC on remyelination and any effect on the demyelination that is expected to occur in the NABT and lesions of patients with RRMS are unknown. The present pilot study was designed to investigate changes in VW-MTR in NABT and lesions in patients with RRMS receiving 24 weeks of treatment with IFN β-1a SC in comparison with untreated, healthy controls (HCs).

### Objectives

The primary objective was to characterize the effect of 24 weeks of treatment with IFN β-1a SC on VW-MTR dynamic mapping of NABT in patients with RRMS with reference to HCs.

Secondary objectives were to characterize the effect of treatment on VW-MTR dynamic mapping of T2, T1 and Gd-enhancing lesions in patients over various treatment periods (baseline to 12 weeks, 12 to 24 weeks, baseline to 24 weeks), and with reference to HCs. The effect of treatment on VW-MTR dynamic mapping of NABT and lesions on the first 12 weeks (baseline to 12 weeks) compared with the second 12 weeks (12 to 24 weeks) was also measured in patients and HCs.

## Methods

The protocol for this trial and CONSORT checklist are available as Checklist S1 and Protocol S1. A 24-week, open-label, two-arm, single-center pilot trial (ClinicalTrials.gov: NCT01085318) was conducted in patients with RRMS and in HCs. Subjects gave written informed consent before participation in the study. The trial protocol and all major amendments were approved by the relevant Institutional Review Boards or Independent Ethics Committees and by Health Authorities. The trial was conducted in accordance with the protocol, the International Conference on Harmonization guideline for Good Clinical Practice, and applicable local regulations as well as with the Declaration of Helsinki. The protocol was approved by the University at Buffalo Health Sciences Institutional Review Board.

Screening was performed within 14 days prior to study entry. Baseline assessments included physical and neurologic exams, MRI scans and laboratory studies. Study patients had brain MRI scans on Study Day 1, and at 12 and 24 weeks, with neurologic examinations conducted at screening and at 12 and 24 weeks. The MRI analysis was rater-blinded. Neurologic examinations were not blinded. A safety evaluation telephone call was scheduled 4 weeks post study exit to monitor the subjects’ status.

The main inclusion criteria were: age 18–65 years; diagnosis of MS according to the revised 2010 McDonald criteria [21]; RRMS disease course [22], treatment-naïve or currently using any of the US Food and Drug Administration-approved disease-modifying drugs; and disease duration of ≤20 years. Main exclusion criteria were: plasmapheresis within 3 months prior to screening; treatment with immunosuppressant agents within 30 days prior to screening; any other concomitant immunomodulatory therapies; relapse within 30 days prior to screening; steroid treatment within 30 days prior to MRI on Study Day 1; alanine aminotransferase >2.5×upper limit of normal (ULN), alkaline phosphatase >2.5×ULN or total bilirubin >1.5×ULN; total white blood cell count <3.0×10^9^/L, platelet count <75×10^9^/L or hemoglobin <100 g/L; complete transverse myelitis or simultaneous-onset bilateral optic neuritis; thyroid dysfunction; moderate to severe renal impairment; history of seizures not adequately controlled by treatment; and serious or acute cardiac disease.

Patients received IFN β-1a SC tiw for 24 weeks, titrated over 4 weeks to a final dose of 44 mcg.

### Data Collection

MRI exams of the brain were performed on a 3T GE Signa LX Excite 12.0 scanner. In addition to localizer/scout images and calibration images, the following scans were acquired: dual echo proton density (PD) and T2-weighted image (WI), 3D-inversion recovery fast-spoiled-gradient recalled (IR-FSPGR), T1-WI, spin-echo (SE) T1-WI with (patients only) and without Gd contrast, fluid attenuated inversion recovery (FLAIR), PD-weighted (used as the magnetization transfer unsaturated M_0_ image) and PD-weighted with magnetization transfer (M_S_). Conventional 2D scans (PD/T2, FLAIR and T1 SE pre- and post-contrast) were acquired with consistent voxel size (48 slices of 3 mm slice thickness, with FOV = 25.6, matrix = 256×256 and phase FOV = .75), and slice selection (oblique axial images parallel to the subcallosal plane).The PD M_0_ was acquired with 3 mm slice thickness, FOV = 25.6, phase FOV = 0.75, matrix 256×256, TR = 2400, TE = 22 and FA = 90. The PD M_S_ was acquired identically to the M_0_ except for the addition of a magnetization transfer pulse with an offset of 1200 Hz. Patients received a single dose (0.1 mmol/kg) of Gd contrast. HCs did not receive contrast injection. All participants were positioned in the scanner to maximize comfort and minimize head movement. The nasion landmark was used to position the head in the isocenter of the magnetic field.

For the primary endpoint, the volumes of NABT with increasing or decreasing in VW-MTR value from baseline to 24 weeks in patients treated with IFN β-1a SC were compared with changes in VW-MTR in NABT of HCs. Secondary endpoints were the volumes of tissue significantly increasing and significantly decreasing in VW-MTR value from baseline to 12 weeks and from 12 to 24 weeks in NABT, and increasing and decreasing VW-MTR values at 24 weeks versus baseline in T2, T1 and Gd-enhancing lesions.

The VW-MTR analysis was previously described in detail [7]. Briefly, the following processes were applied: 1) Lesions were identified on T2/PD/FLAIR-WI, T1 pre-contrast (black holes) WI and T1 post-contrast (Gd-enhancing) WI, as previously described [23]; 2) VW-MTR and accompanying T1-WI, T1+Gd-WI, T2/PD/FLAIR-WI and 3D-T1-WI images were co-registered using FLIRT (FMRIB, Oxford, UK); 3) 3DT1-WI were segmented to classify voxels as gray matter, white matter and cerebrospinal fluid, as well as to eliminate voxels with significant partial volume changes. Misclassification of T1 hypointensities were avoided using an in-house-developed inpainting technique; 4) VW-MTR maps were generated by a voxel-wise application of the standard MTR formula ([(M_0_–M_S_)/M_0_]×100); 5) VW-MTR difference maps were created by subtracting VW-MTR map pairs based on longitudinal time points; 6) Threshold-free cluster enhancement (TFCE) was applied to the difference maps to increase classification sensitivity; 7) A Monte Carlo process was used to derive statistically rigorous probability values associated with the TFCE-generated voxel clusters; 8) Voxels were classified as increasing or decreasing in NABT, T2, T1 and T1+Gd lesion volumes and 9) Image quality control was performed at every stage, beginning with the quality control of the raw input images.

Frequency and number of adverse events (AEs) in HCs and patients over the duration of the study were recorded.

### Statistical Methods

A total of 40 subjects, comprising 25 patients and 15 HCs, were planned to be enrolled.

All statistical tests were two-tailed and considered significant at the α = 0.05 level of significance. Student’s t-tests and Fisher’s exact tests were used to test for differences in demographic and clinical characteristics between patients and HCs. Non-parametric tests were used for comparisons of MRI data, since MRI data were not expected to be normally distributed. The Wilcoxon rank–sum test was used for testing the difference between patients and HCs. The Wilcoxon signed–rank test was used for testing changes from baseline within study groups and differences across time (i.e., first 12 weeks versus second 12 weeks of the study) within study groups. If lesion volumes in HCs were not measurable, only comparisons within the patient group were made. With the exception of the primary endpoint, which was not corrected?, multiple comparisons were corrected using the Holm-Bonferroni method.

## Results

### Study Participants

**Figure 1:**
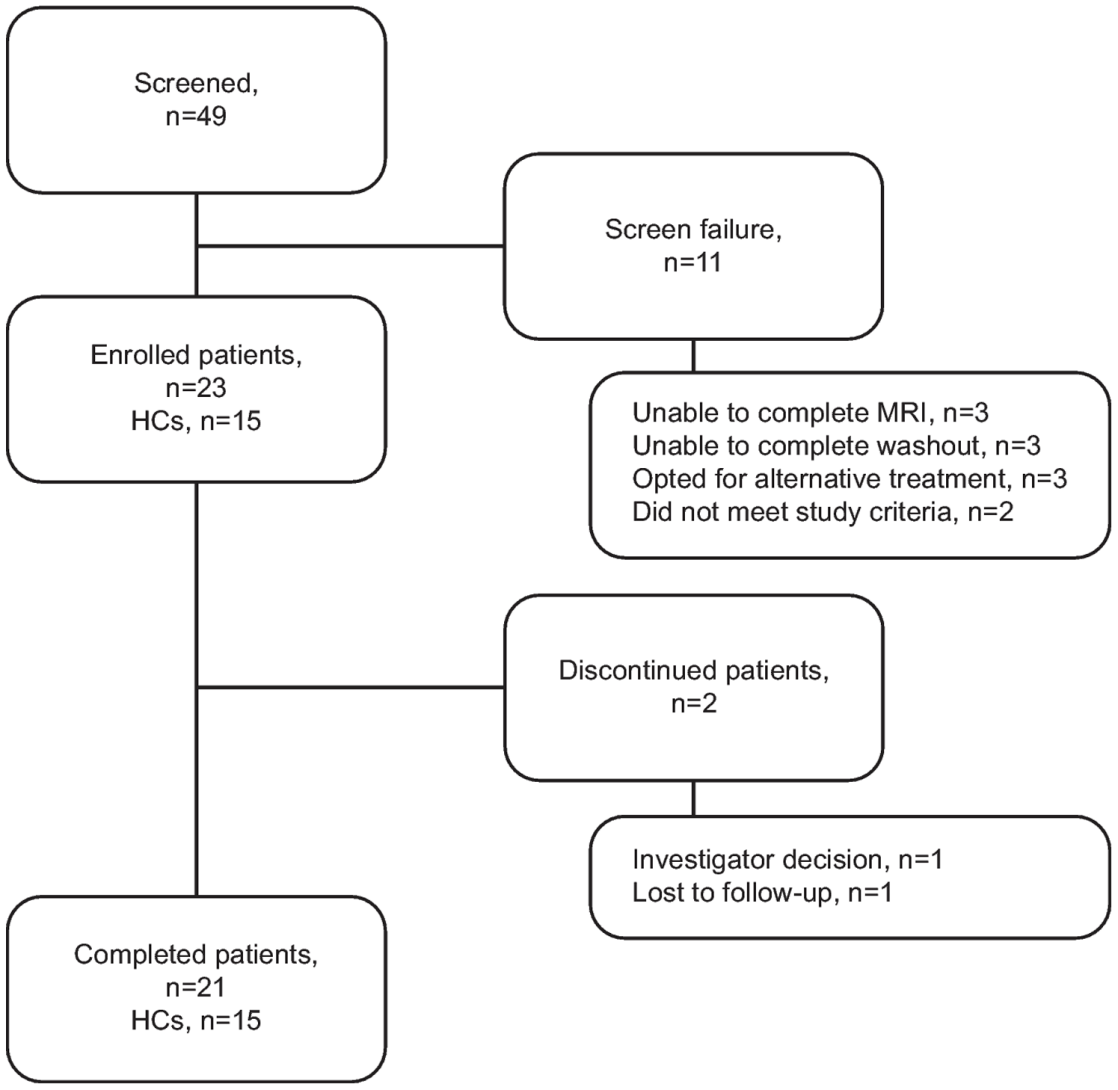
Subject numbers from enrollment to study completion. HC, healthy control; MRI, magnetic resonance imaging.

Twenty one/23 enrolled patients and all 15 HCs completed the study. Reasons for discontinuations are recorded in Figure 1. Baseline characteristics of patients and HCs (including history of pre-study therapies) are shown in Table 1. There were no significant differences in demographic characteristics between the study groups. Study drug exposure and compliance during the study is shown in Table 1.

**Table 1:** Baseline characteristics of patients and of HCs and study drug exposure and compliance throughout the study.

Baseline characteristic	Patients n = 23	HCs n = 15	p-value for between-group comparison
Age, years, mean (SD)	39.9 (10.17)	36.7 (10.31)	0.362^a^
Female, n (%)	14 (61)	8 (53)	0.743^b^
Race, n (%)			0.144^b^
White	20 (87)	14^c^ (93)	
Black	3 (13)	0	
Other: Indian	0	1 (7)	
Weight, kg, mean (SD)	79.9 (22.25)	87.0 (18.33)	0.313^a^
Height, cm, mean (SD)	171.0 (8.48)	168.5 (6.99)	0.349^a^
BMI, kg/m^2^, mean (SD)	27.2 (6.90)	30.5 (5.37)	0.128^a^
MS history	**Patients n = 23**		
Years since MS diagnosis, mean (SD), range	6.6 (5.65), 0–20		
Years since most recent relapse, mean (SD), range	1.0 (1.14), 0.1–5.0		
Number of relapses in past 12 months,^d^ mean (SD)	1.26 (1.18)		
0	7 (30%)		
1	7 (30%)		
2	7 (30%)		
4	2 (9%)		
Most recent DMD use, n (%)			
Avonex	8 (35)		
Rebif	5 (22)		
Copaxone	4 (17)		
Tysabri	2 (9)		
IV Ig	1 (4)		
None	3 (13)		
EDSS score, median (range)	2.5 (1.0–5.5)		
Ambulation distance, meters, mean (SD)	475 (94.2)		
**Study drug exposure and compliance** e	**IFN β-1a SC tiw n = 23**		
	**8.8 mcg**	**22 mcg**	**44 mcg**
Total dose received, mcg, mean (SD)	52.5 (7.60)	133.9 (9.17)	2174.9 (584.47)
Doses missed, mean (SD)	0 (0.2)	0 (0)	5 (7.6)
Compliance, %, mean (SD)	99 (3.5)	100 (0)	92 12.2)

a For the difference between groups from the Student’s *t*-test.

b For the difference between groups from Fisher’s exact test.

c Includes one of Hispanic ethnicity, all other subjects were not Hispanic.

d Patients reported the same number of relapses for the past 24 months.

e Compliance was calculated at study visits using subject diaries and returned drug. Exposure was calculated from compliance.

BMI; body mass index; DMD, disease-modifying drug; EDSS, Expanded Disability Status Scale; HC, healthy control; IFN β-1a, interferon beta-1a; IV Ig, intravenous immunoglobulin; MS, multiple sclerosis; SC, subcutaneous; SD, standard deviation; tiw, three times weekly.

Between 5 and 16 patients scored as abnormal on various items of the neurologic assessments. Most HCs scored as normal, although 2 two HCs scored as abnormal on cranial nerve status and 1 HC scored as abnormal on reflex and sensory status.

Baseline MRI T2, T1 and Gd-enhancing lesion assessment data are shown in Table 2. Eight/23 patients (35%) had ≥1 Gd-enhancing lesion at baseline; patient ID #29 had the greatest number of Gd-enhancing lesions.

**Table 2:** Baseline MRI T2, T1 and Gd-enhancing lesion assessments.

	Patients n = 23	HCs n = 15
**T2 lesions**		
Number, mean (SD)	34.9 (24.51)	0.8 (1.37)
Median (range)	28 (5–115)	0 (0–4)
Volume, mm^3^, mean (SD)	23683 (27731)	57 (151)
Median (range)	17739 (558–118940)	0 (0–588)
**T1 lesions**		
Number, mean (SD)	19.5 (15.34)	0 (0)
Median (range)	19 (0–59)	0 (−)
Volume, mm^3^, mean (SD)	4532 (5417)	0 (0)
Median (range)	2847 (0–23364)	0 (−)
**Gd-enhancing lesions**		
Number, mean (SD)	1.8 (4.84)	–
Median (range)	0 (0–22)	–
Volume, mm^3^, mean (SD)	240 (646)	–
Median (range)	0 (0–2915)	–
**Patient ID #/number of lesions**		
29	22	
03	9	
22	4	
13, 23	2	
07, 08, 18	1	
All others	0	

Gd, gadolinium; HC, healthy control; MRI, magnetic resonance imaging; SD, standard deviation.

### Change in Volume of NABT with VW-MTR Following 12 and 24 Weeks of Treatment with SC IFN β-1a

**Figure 2:**
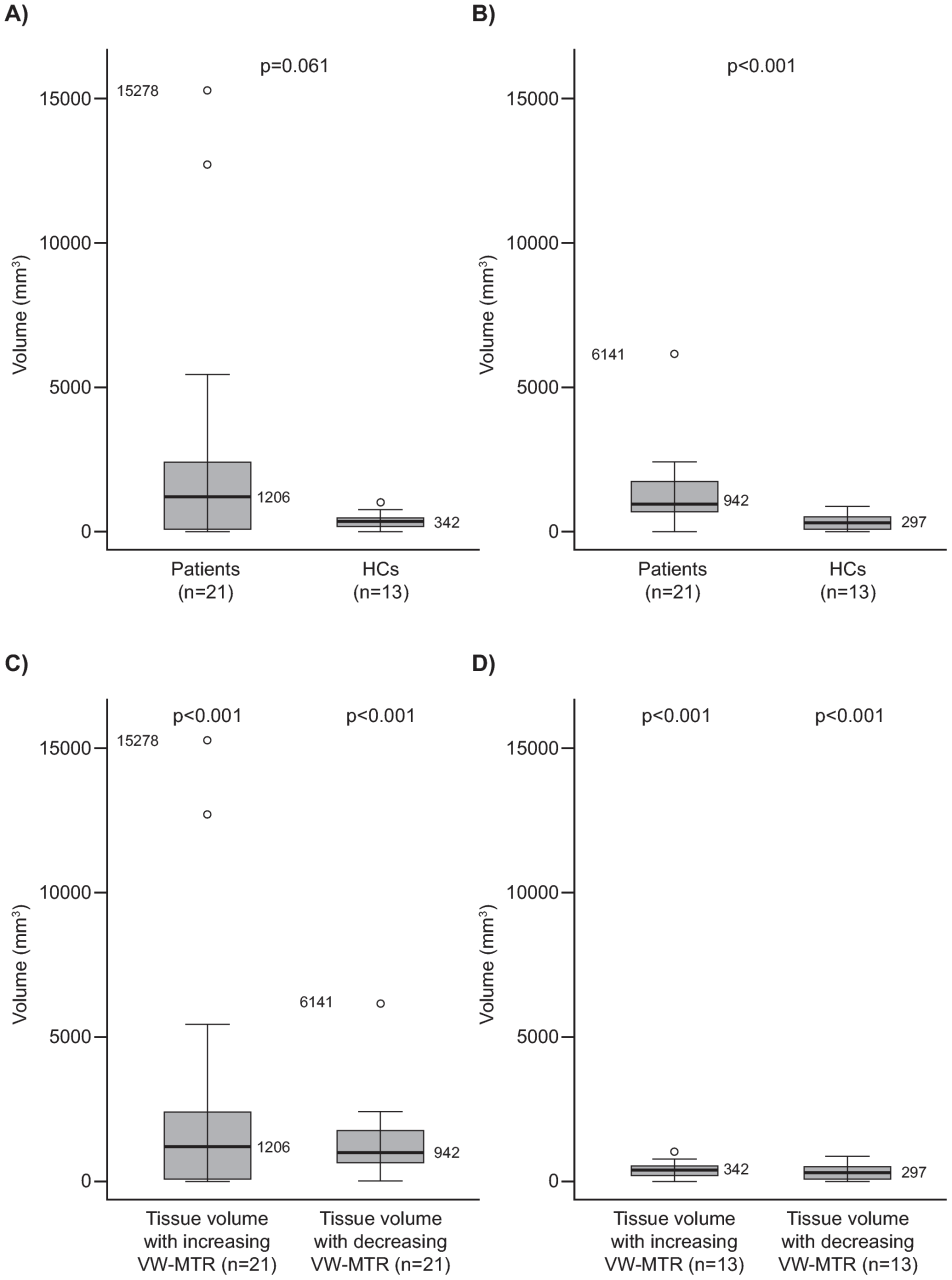
Change in VW-MTR in NABT from baseline to 24 weeks. The figure depicts a comparison between patients and HCs with A) increasing VW-MTR and B) decreasing VW-MTR and difference from baseline within each study group for increasing and decreasing VW-MTR in C) patients and D) HCs. For A) and B), the p value is the difference between patients and HCs from the Wilcoxon rank–sum test; for C) and D), the p-value is the difference from baseline within groups from the Wilcoxon signed–rank test. In the box plots, the bold line represents the median; the boxes represent the middle 50% of data; the top and bottom of the box represent the third and first quartiles; the open circles are outliers. The whisker lines above and below the boxes represent the largest and smallest values that are not considered to be outliers. HC, healthy control; NABT, normal appearing brain tissue; VW-MTR, voxel-wise magnetization transfer ratio.

The median [range] difference in NABT volume with increase in VW-MTR from baseline to 24 weeks between patients and HCs approached significance (1206 [0–15278] and 342 [0–951] mm^3^, respectively; p = 0.061, unadjusted) (Figure 2A and Table 3). From baseline to 12 weeks, there was a significant change in NABT volume with increasing VW-MTR in patients compared with HCs (852 [6–11577] and 360 [0–1755] mm^3^, respectively; p = 0.028, unadjusted) (Table 3).

**Table 3:** Change in VW-MTR in NABT over time – comparison between patients and HCs with increasing and decreasing VW-MTR (mm^3^).

	Baseline to 12 weeks		12 to 24 weeks		Baseline to 24 weeks	
	Patients	HCs	Patients	HCs	Patients	HCs
n (missing^a^)	22 (1)	15 (0)	20 (3)	13 (2)	21 (2)	13 (2)
**Increasing VW-MTR (mm^3^)**						
Mean (SD)	1467 (2482)	445 (474)	1161 (1325)	420 (459)	2473 (4082)	356 (292)
Median	852	360	764	201	1206	342
Min, Max	6, 11577	0, 1755	0, 4518	36, 1239	0, 15278	0, 951
p-value^b^	<0.001	<0.001	<0.001	<0.001	<0.001	<0.001
Adjusted p-value^b^^,^ c	<0.001	<0.001	<0.001	0.001	<0.001	0.002
p-value^d^	0.028		NS		0.061	
Adjusted p-value^c^^,^ d	NS		NS		–	
Adjusted p-value^c^^,^ e	NS/NS					
**Decreasing VW-MTR (mm^3^)**						
Mean (SD)	1349 (1076)	274 (237)	1513 (1974)	388 (349)	1346 (1263)	358 (311)
Median	1092	186	779	279	942	297
Min, Max	279, 4321	3, 822	156, 8499	57, 1263	0, 6141	0, 852
p-value^b^	<0.001	<0.001	<0.001	<0.001	<0.001	<0.001
Adjusted p-value^b^^,^ c	<0.001	<0.001	<0.001	0.001	<0.001	0.001
p-value^d^	<0.001		0.006		<0.001	
Adjusted p-value^c^^,^ d	<0.001		NS		–	
Adjusted p-value^c^^,^ e	NS/NS					

a Failed analysis or missing MRI at Week 24.

b For the difference from zero within groups from the Wilcoxon signed–rank test.

c Adjusted for multiple comparisons with the Holm-Bonferroni correction.

d For the difference between IFN β-1a SC and HC from the Wilcoxon rank–sum test.

e For the difference between the first 12 weeks and the second 12 weeks within groups from the Wilcoxon signed–rank test (p-value for patients/HCs).

HC, healthy control; IFN β-1a, interferon beta-1a; NABT, normal appearing brain tissue; Max, maximum; Min, minimum; NS, non-significant; SC, subcutaneously; SD, standard deviation; VW-MTR, voxel-wise magnetic transfer ratio.

Change in NABT volume with decreasing VW-MTR from baseline to 24 weeks was significantly greater in patients compared with HCs (942 [0–6141] and 297 [0–852] mm^3^; p<0.001, unadjusted (Figure 2B), and at other study timepoints (Table 3). The median [range] difference in volume of NABT with decreasing VW-MTR in NABT between patients and HCs (1092 [279–4321]; 186 [3–822] mm^3^, respectively) was also significant (p<0.001, adjusted) from baseline to 12 weeks, but not significant from 12 to 24 weeks following adjustment (Table 3).

Within the patient group, there was a tendency for a greater inter-subject variation in volume of NABT with increasing and decreasing VW-MTR (Figure 2C), while HCs had very similar, low-level increasing and decreasing VW-MTR (Figure 2D).

**Figure 3:**
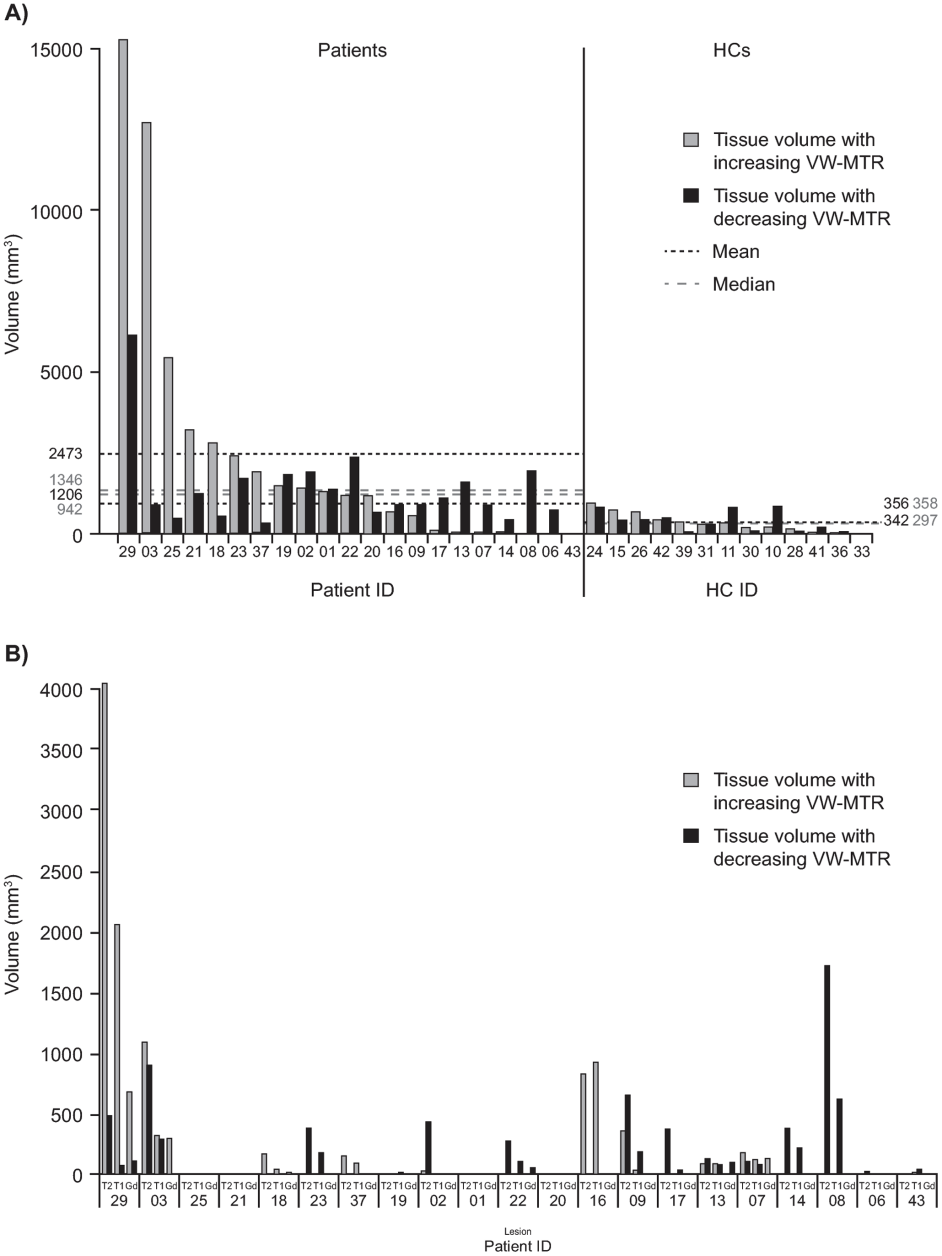
Change in VW-MTR from baseline to 24 weeks. Change in A) NABT in patients and in HCs, B) T2-LV, T1-LV and Gd-enhancing lesions in patients only. Gd, gadolinium*;* HC, healthy control; LV, lesion volume; NABT, normal appearing brain tissue; VW-MTR, voxel-wise magnetization transfer ratio.

In 2 patients, markedly increased VW-MTR volumes in NABT were observed compared with all other patients (15278 and 12000 mm^3^) (Figure 3A); these patients also had the highest number of Gd-enhancing lesions (patient ID #s 29 and 03) at baseline (Table 2). One patient appeared not to have any measurable changes in VW-MTR (Figure 3A).

### Change in VW-MTR Lesion Volume Following 24 Weeks of Treatment with IFN β-1a SC

#### T2-LV

**Figure 4:**
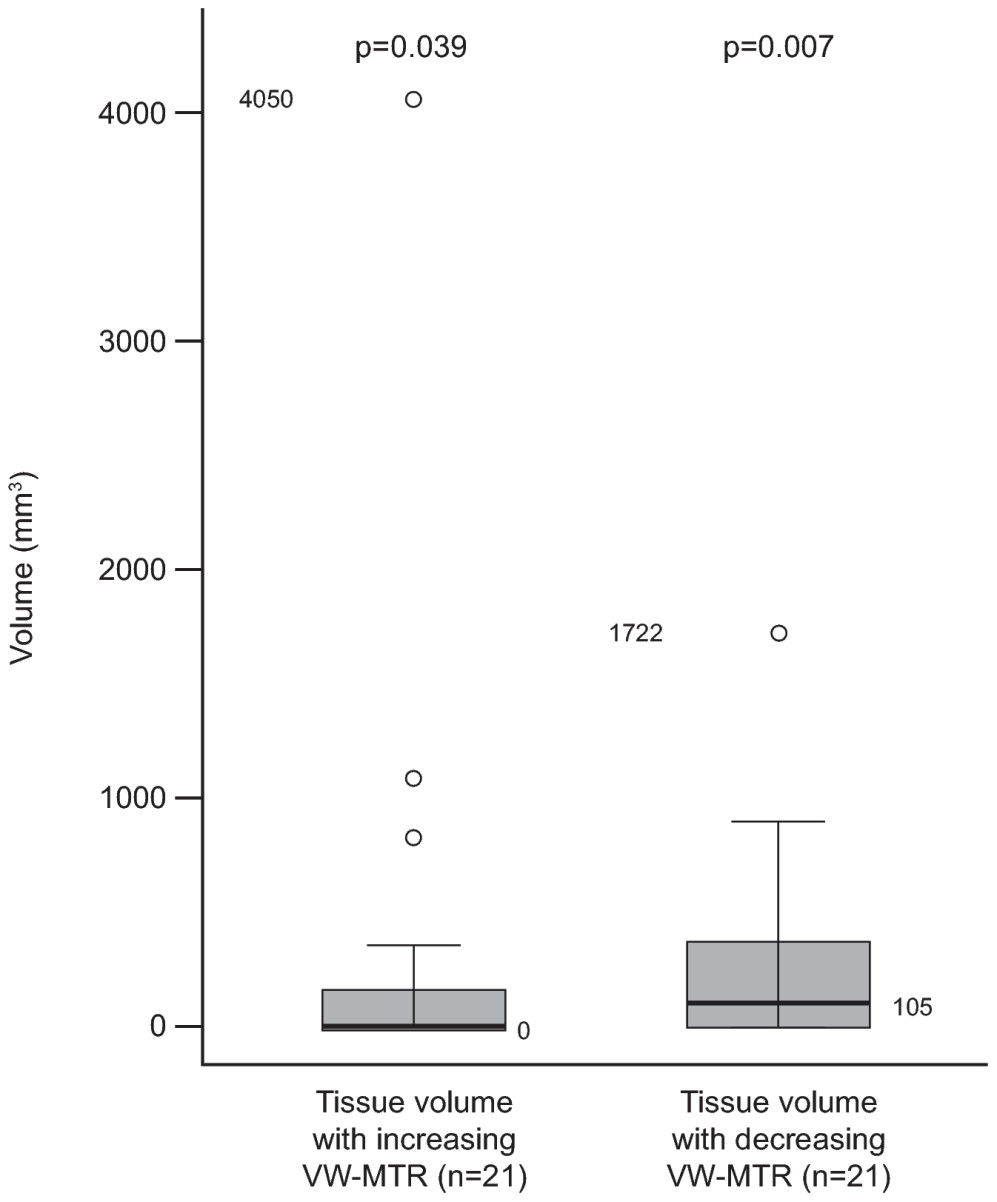
Difference from baseline to 24 weeks for increasing and decreasing VW-MTR in T2-LV in patients. The p-value is for the difference from zero within groups from the Wilcoxon signed–rank test with adjustment. In the box plot, the bold line represents the median; the boxes represent the middle 50% of data; the top and bottom of the box represent the third and first quartiles; the open circles are outliers. The whisker lines above and below the boxes represent the largest and smallest values that are not considered to be outliers. LV, lesion volume; NS, not significant; VW-MTR, voxel-wise magnetization transfer ratio.

The change in T2-LV was 0 for baseline to 12 weeks and for baseline to 24 weeks for all HCs. A change in T2-LV with increasing VW-MTR of 18 mm^3^ was noted from 12 to 24 weeks in a single HC (ID #11). Changes in T2-LV with increasing and decreasing VW-MTR in patients are summarized in Table 4. Although the median change in T2-LV with increasing VW-MTR from baseline to 24 weeks for patients was 0, there was a significant change from baseline for this group (median [range] of 0 [0–4050] mm^3^; p = 0.039; adjusted for multiple comparisons) (Figure 4). The change in T2-LV with decreasing VW-MTR from baseline to 24 weeks was also significant in patients (median [range] of 105 [0–1722] mm^3^; p = 0.007, adjusted for multiple comparisons) (Figure 4). There was a greater degree of change occurring for T2-LV with decreasing VW-MTR than with increasing VW-MTR in patients. One patient (patient ID # 29) appeared to have a very high (4050 mm^3^) amount of increasing VW-MTR. Outliers in T2-LV with decreasing VW-MTR were not as pronounced, with an outlier (patient ID # 08) at 1722 mm^3^. Six patients did not appear to have any T2-LV with increasing or decreasing VW-MTR; whereas 6 patients had both; 3 patients had T2-LV with increasing VW-MTR only and 6 patients had T2-LV with decreasing VW-MTR only (Figure 3B).

**Table 4:** Change in T2-LV and T1-LV over time in patients with increasing and decreasing VW-MTR (mm^3^).

	T2-LV			T1-LV		
	Baseline to 12 weeks	12 to 24 weeks	Baseline to 24 weeks	Baseline to 12 weeks	12 to 24 weeks	Baseline to 24 weeks
n (missing^a^)	22 (1)	20 (3)	21 (2)	22 (1)	20 (3)	21 (2)
**Increasing VW-MTR (mm^3^)**						
Mean (SD)	189 (641)	78 (225)	330 (900)	114 (430)	63 (206)	176 (478)
Median	3	0	0	0	0	0
Min, Max	0, 3033	0, 1008	0, 4050	0, 2022	0, 924	0, 2058
p-value^b^	<0.001	0.004	0.004	0.008	0.063	0.004
Adjusted p-value^b^^,^ c	0.012	0.043	0.039	NS	NS	0.035
Adjusted p-value^c^^,^ d	NS		–	NS		–
**Decreasing VW-MTR (mm^3^)**						
Mean (SD)	228 (594)	127 (202)	278 (420)	95 (245)	61 (113)	91 (149)
Median	149	27	105	45	0	33
Min, Max	0, 2844	0, 786	0, 1722	0, 1173	0, 456	0, 621
p-value^b^	<0.001	<0.001	<0.001	<0.001	0.004	<0.001
Adjusted p-value^b^^,^ c	0.002	0.004	0.007	0.007	0.031	0.006
Adjusted p-value^c^^,^ d	NS		–	NS		–

a Failed analysis or missing MRI at Week 24.

b For the difference from zero within groups from the Wilcoxon signed–rank test.

c Adjusted for multiple comparisons with the Holm-Bonferroni correction.

d For the difference between the first 12 weeks and the second 12 weeks from the Wilcoxon signed–rank test.

LV, lesion volume; Max, maximum; Min, minimum; MRI, magnetic resonance imaging; NS, non-significant; SD, standard deviation; VW-MTR, voxel-wise magnetic transfer ratio.

#### T1-LV

**Figure 5:**
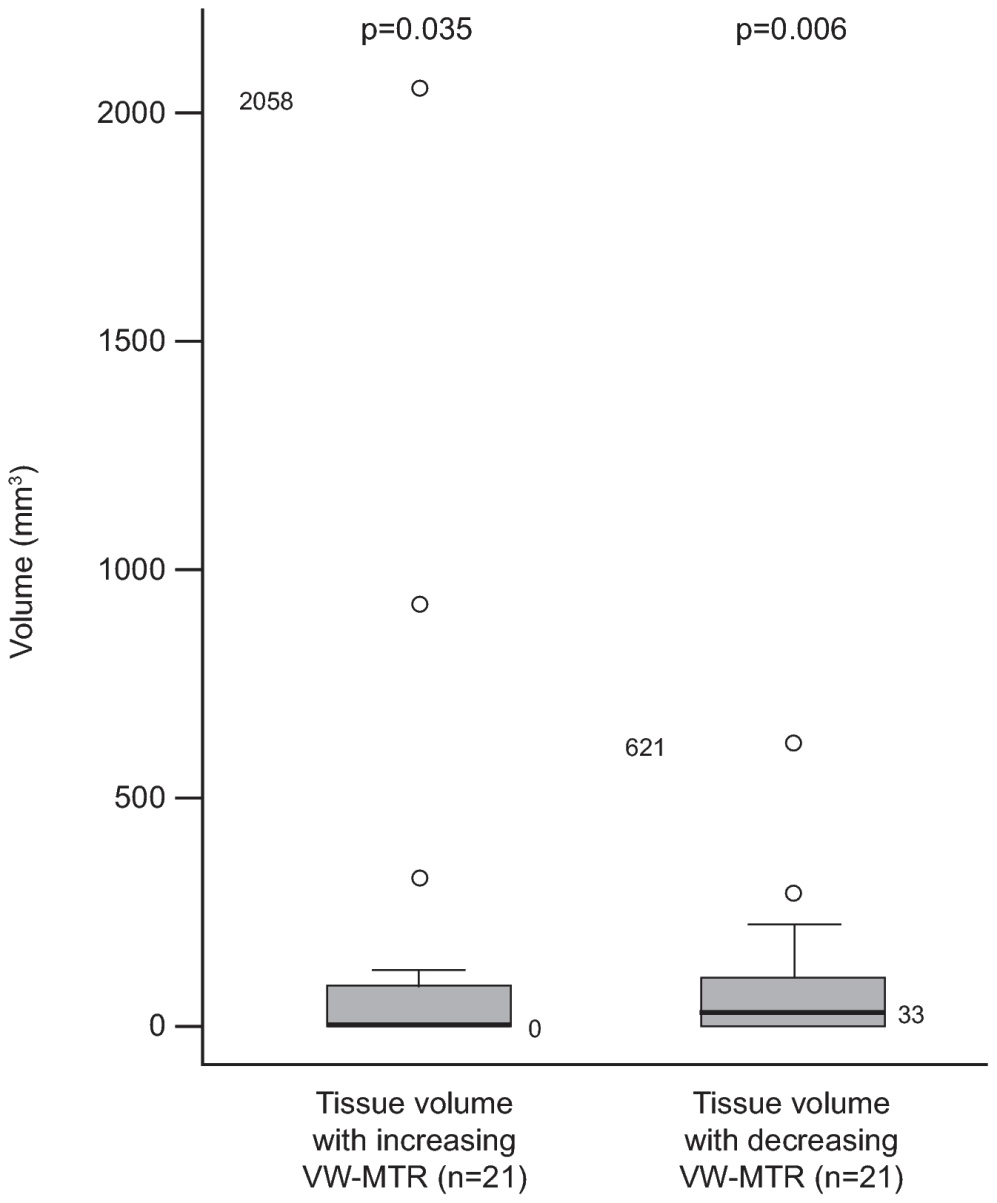
Difference from baseline to 24 weeks for increasing and decreasing VW-MTR in T1-LV in patients. The p-value is for the difference from zero within groups from the Wilcoxon signed–rank test with adjustment. In the box plots, the bold line represents the median; the boxes represent the middle 50% of data; the top and bottom of the box represent the third and first quartiles; the open circles are outliers. The whisker lines above and below the boxes represent the largest and smallest values that are not considered to be outliers. LV, lesion volume; VW-MTR, voxel-wise magnetization transfer ratio.

There was no change in T1-LV for all HCs for all timepoint comparisons. Changes in T1-LV with increasing and decreasing VW-MTR in patients are summarized in Table 4. Similar to T2-LV, the median change in T1-LV with increasing VW-MTR from baseline to 24 weeks for patients was 0, yet there was a significant change from baseline for this group (median [range] of 0 [0–2058] mm^3^; p = 0.035; adjusted for multiple comparisons [Figure 5]; mean [standard deviation (SD)] increase 176 [478] mm^3^.).

Although the distributions of increasing and decreasing VW-MTR for T1-LV for baseline to 24 weeks are more similar than they were for T2-LV, more extreme outliers were again observed in T1-LV for increasing VW-MTR than for decreasing VW-MTR. Patient ID # 29 had an outlier volume of increasing VW-MTR of 2058 mm^3^ and a decreasing volume of VW-MTR of 72 mm^3^ in T1-LV. T1-LV with increasing VW-MTR was not detected in the patient with the greatest volume of decreasing VW-MTR in T1 lesions (621 mm^3^ in patient ID # 08; Figure 3B). As with T2-LV, 6 patients did not appear to have any volumes of increasing or decreasing VW-MTR in T1-LV from baseline to 24 weeks, whereas 6 patients had both, 3 patients had T1-LV with increasing VW-MTR only and 6 patients had T1-LV with decreasing VW-MTR only.

#### Gd-enhancing-LV

For HCs, measurement of Gd-enhancing LV was not performed. There were no significant changes from baseline of either increasing or decreasing VW-MTR in Gd-enhancing LV. Of the 8 patients who had Gd-enhancing lesions at baseline, 2 patients had notably higher amounts of increasing VW-MTR compared with other patients (patient ID #29 and 03). Fifteen patients had neither increasing nor decreasing VW-MTR in Gd- enhancing LV from baseline to 24 weeks; increasing and decreasing VW-MTR of Gd-enhancing LV were both observed in only 1 patient (patient ID # 29). Three patients had only increasing VW-MTR in Gd-enhancing LV and 2 patients had only decreasing VW-MTR (Figure 3B).

### Differences in VW-MTR across Time Points

The differences in volume of NABT, T2 and T1 lesions with increasing VW-MTR between the first 12 weeks and the second 12 weeks of the study in patients were not significant following adjustment for multiple comparisons (Tables 3 and 4). This was also the case for decreasing VW-MTR.

### Relapses

In total, 5/23 (22%) patients had eight relapses; 4 patients (ID #s 09, 17, 23 and 43) each had one relapse of moderate severity and 1 patient (ID # 07) had four relapses, three of moderate severity and one of mild severity (recorded as a serious AE following exposure to treatment) (Table 5).

**Table 5:** Relapses occurring in patients during the study.

	Patients n = 23
**Time to first relapse months, mean (SD), range**	2.5 (1.88), 0–4 months
**Patient ID #/number of relapses**	
09	1
17	1
23	1
43	1
07	4

SD, standard deviation.

### Safety

The majority of patients (22/23) had ≥1 AE; 16 patients were considered to have had AEs related to study treatment. Frequencies of AEs are summarized in Table 6. The most commonly occurring (≥3) treatment-emergent AEs in the patients included influenza-like illness and injection-site reaction.

**Table 6:** Adverse event summary.

n (%)	Patients n = 23	HCs n = 15
≥1 AE	22 (96)	8 (53)
≥1 treatment-related AE^a^	16 (70)	0
≥1 severe AE	4 (17)	0
≥1 serious AE	1 (4)	0
≥1 AE resulting in discontinuation of study drug^b^	1 (4)	0
**Most common (≥3) TEAEs, n (%)**	**Patients n = 23**	
Influenza-like illness	9 (39)	
Injection-site reaction	8 (35)	
Multiple sclerosis relapse	5 (22)	
Fatigue	4 (17)	
Injection-site pain	4 (17)	
Nasopharyngitis	4 (17)	
Headache	3 (13)	
Migraine	3 (13)	
Muscular weakness	3 (13)	
Nausea	3 (13)	

a Relationship = possible, probable.

b Action taken = medication permanently stopped.

AE; adverse event; HC, healthy control; TEAE, treatment-emergent AE.

## Discussion

The primary objective of this pilot study was to investigate the effects of IFN β-1a SC treatment over 24 weeks on VW-MTR volume changes in NABT in patients with RRMS. Secondary objectives were the measurement of changes in VW-MTR LV over 24 weeks and in NABT from baseline to 12 weeks and from 12 to 24 weeks following treatment with IFN β-1a SC, as well as safety.

The increases and decreases in VW-MTR observed in the study serve as indications of possible remyelinating and demyelinating processes occurring in NABT, T2, T1 and Gd-enhancing lesions of patients [7]. For NABT, increases in VW-MTR in patients were much greater than those observed in HCs, but the variation was such that the difference fell short of significance at 24 weeks. It is possible that loss of statistical power by 24 weeks contributed to this result, as MRI data for 2 patients and 2 HCs were not available for the Week 24 comparison, thus reducing the already small sample size available for statistical analysis. From baseline to 12 weeks, NABT tissue volume with increasing VW-MTR was significantly greater in patients compared with HCs (unadjusted p-value), which could suggest that IFN β-1a SC treatment may be associated with an early surge in remyelinating activity. This is consistent with the effect of type I IFNs suggested in a virus-infected SJL/J mouse model of MS (Theiler’s murine encephalomyelitis), in which short-term treatment (5 weeks) led to a significant increase in remyelinated spinal cord white matter [24].

When the data from individual patients were analyzed, the large variations observed are attributed to very high increasing VW-MTR volumes in 3 patients (ID #s 29, 03, 25). Similarly, for T2 lesions, increases in VW-MTR appeared to be driven by a small number of patients. The sensitivity of the VW-MTR imaging technique enabled the distinction of this subpopulation of patients, and suggested that remyelination in these patients was very prominent. However, the results need to be verified by a larger study.

The between-patient variability in tissue volume with changing VW-MTR is consistent with previous VW-MTR studies [8]. A profound diversity in the amount of remyelination between cases has also been observed by immunohistochemistry, with evidence of extensive remyelination occurring in RRMS and progressive disease [14]. As histopathological analysis of a Gd-enhancing lesion has been shown to correlate with VW-MTR [11], further validation of VW-MTR extrapolation to remyelination and demyelination by histopathology may help better define this relationship [25].

Baseline disease activity may affect treatment response; having a high number of Gd-enhancing lesions was expected to result in little increasing VW-MTR activity in NABT [11]. However, the 2 patients (ID #s 29, 03) with the greatest number of Gd-enhancing lesions at baseline (22 and 9 lesions, respectively) had markedly increased VW-MTR changes in NABT, suggesting that higher inflammatory activity may correlate with much higher remyelination. From this study, definitive conclusions cannot be drawn as to whether IFN β-1a SC treatment caused the observed higher remyelination, as untreated patients were not studied. However, the results are intriguing and it is interesting to speculate that IFN β-1a SC may be exerting an anti-inflammatory effect, which is then influencing the remyelination process. Investigations of correlations between the effect of IFN β-1a SC on Gd-enhancing lesions and VW-MTR changes would be interesting. An explanation for those patients where increasing VW-MTR activity was sparse might be that prior repeated demyelinating and remyelinating processes within lesions could have destroyed oligodendrocyte progenitor cells, leading to inhibition of progenitor cell maturation [26], [27] or axonal inhibition of remyelination [28].

Of the 5 patients who had a relapse during the study, VW-MTR changes in NABT and LV were moderate to small, or negligible in magnitude, relative to patients without relapse. Generally, decreasing VW-MTR appeared to be more prominent than increasing VW-MTR in the 4 patients who each had one relapse. For the patient with four relapses (ID # 07), VW-MTR changes were small; notably, decreasing VW-MTR was dominant over increasing VW-MTR in NABT, while increasing VW-MTR was greater than decreasing VW-MTR in T2-LV, T1-LV and Gd-enhancing LV.

As expected, decreasing VW-MTR in NABT measured versus baseline at 12 and 24 weeks and in T2-LV at 24 weeks was significantly greater in patients than in HCs, supporting the validity and sensitivity of the VW-MTR imaging technique for measuring ongoing demyelinating processes in the RRMS disease state.

### Factors Affecting Measurement of Myelin Content by VW-MTR

The significant changes from baseline to 24 weeks in VW-MTR activity in NABT volume within both the patient and HC groups and in T2-LV and T1-LV within the patient group are suggestive of dynamic shifts occurring in myelin content. It then follows that a numerically greater degree of change in VW-MTR signal occurred in patients than in HCs in all areas measured is suggestive of greater dynamic shifts in myelin content. The reasons for this are unknown but are assumed to be due to the RRMS disease state, although, without an untreated control RRMS group, an effect due to treatment with IFN β-1a SC cannot be ruled out. It has to be noted that subtle movement artifacts may be an important confounding source in the VW-MTR analysis. However, we believe we have adequately controlled for this in two ways. First, motion artifacts tend to create high-frequency, disconnected “ridges”, which are not generally picked up by the TFCE approach that we used to preprocess the data. Second, and more directly, we specifically included motion artifacts in our quality control checks, and manually removed them if detected.

The VW-MTR imaging technique has advantages over conventional MRI because its voxel-wise approach to image processing avoids the definition of *a priori* ROIs inherent to other MRI approaches, thereby reducing background noise and increasing sensitivity to a level capable of reporting on more subtle or heterogeneous tissue changes (e.g., myelination processes in NABT) not normally detectable by ROI-based approaches. On the other hand, potential confounding influences on the sensitivity of VW-MTR to myelination processes in lesions may result from rapid, transient changes in re- and demyelination events and to the associated edema that accompanies inflammation during the acute period of lesion formation. In the brains of 7 patients with MS, significant correlation was found between water content and MTR, indicating that inflammation and edema influence MTR in those patients, and so decreasing MTR may not be exclusively due to demyelination [29]. However, post-lesion MTR values appear to stabilize after 6 months [10], giving confidence that mean MTR changes are unlikely to be affected by transient inflammation and edema and better reflect pure myelination processes after this time point. The changes in the NABT VW-MTR due to edema resolution are even less likely after 6 months. Edema itself may correlate poorly with myelin content; the conclusion from an *ex vivo* study in rat spinal cord was that intercompartmental exchange of water between myelin and non-myelin compartments may cause the myelin water fraction to underestimate the true myelin content of tissue. However, MTR metrics appear to be insensitive to intercompartmental water exchange in comparisons with multi-exponential T2 relaxation MRI measures [30]. In an experimental autoimmune encephalomyelitis model [31], MTR and myelin water percentage appeared to measure different aspects of pathology, and modulation of inflammatory activity did not correlate with myelin water percentage.

### Safety

There were no unexpected AEs reported following treatment with IFN β-1a SC, which was well-tolerated by patients in this study. Injection-site reactions and influenza-like illness were, as expected, the most commonly occurring AEs, consistent with previous studies where these events were also noted as common AEs [19], [20], [32].

### Limitations of this Study

The myelin specificity of VW-MTR and the evidence from previous studies suggesting that increases in VW-MTR may be attributed to remyelination in MS [7], [8], [10], [16] should be considered with some caution, as there does not yet appear to be a well-accepted MRI metric of remyelination [33]. The use of VW-MTR as a possible indication of remyelination is still an emerging technique; further validation is required to discount confounding effects as described above that could cause increasing tissue volumes that are detected by VW-MTR.

The potential benefit of IFN β-1a SC on remyelination as measured by VW-MTR in a head-to-head comparison with untreated or comparator-treated patients was not measured. However, the inclusion of a HC group allowed evaluation of this technique for normal variation between individuals, against which changes in VW-MTR could be compared. Use of HCs for comparison with a patient population in prospective longitudinal pilot studies of non-conventional MRI, including VW-MTR, may become an attractive approach in the future for several reasons. First, the ultimate goal of therapy is to normalize patient VW-MTR changes over time or to reduce variability over time, to those observed in HCs over the same time period. HCs also experience brain changes over time and so the notion of arresting disease progression as measured by VW-MTR requires validation by reference to a comparator without disease progression to account for age-related changes. Second, ethical considerations preclude placebo-controlled studies using an established MS treatment versus placebo-treatment in patients with RRMS. An alternative would be to measure VW-MTR prior to starting treatment; however, this is more difficult from a practical and ethical point of view, as the window of switching from old to new treatments is very narrow in patients with MS at present time and the feasibility of enrolling sufficient numbers of patients with MS who were naïve to treatment was deemed to be low.

Another limitation that should be considered in any future study is that transient, intermittent, and/or longer term effects of treatment with IFN β-1a SC may have been missed by the short duration and limited number of VW-MTR measurement timepoints of this pilot study. As already mentioned, early stage remyelination events occurring in lesions that are still active may be unstable [10], so that formation of persistent, remyelinated shadow plaques earlier in the disease time course will be sparse in contrast to what is observed in inactive lesions of later-stage disease [14]. Previously, a 1-year study was extended to a 2-year follow-up due to evidence of the sensitivity of VW-MTR as a means to monitor disease activity in patients with MS [8]. Significant increases and decreases in MTR have been shown to be ongoing for 7 and 33 months, respectively, in a 3-year study on various regions of acute Gd-enhancing lesions [4]. As potential remyelination follows onset of demyelination [14], a future study design with more frequent VW-MTR measurement timepoints to measure the relative time courses of VW-MTR decreases and increases may capture the temporal signature of RRMS. A benefit of a longer-term study measuring sustained remyelinating effects, in addition to gaining insights on whether and how IFN β-1a SC influences re- and demyelination, would be to minimize any bias in interpretation of VW-MTR measurement by transient fluid shifts. As the magnitude of change from baseline to Week 24 is larger than from baseline to Week 12, enrollment of larger patient and HC populations in future studies would allow for adequate power to detect interesting signals, and would also allow for better characterization of the localizations of VW-MTR changes. Given the small sample and relatively short follow-up, the study was not sufficiently powered to perform spatial mapping of changes in a meaningful way. However, in the previous study, we noted possible regional specificity of the localizations of VW-MTR changes with increases in VW-MTR being more confined to the frontal lobes and the decreases in VW-MTR being more confined to the parietal and occipital lobes [8].

## Conclusions

A significant change in NABT volume with increasing VW-MTR was observed in patients with RRMS following treatment with IFN β-1a SC tiw at 12 weeks. Further studies are required to investigate this as a possible treatment effect. This study further validates VW-MTR as a potentially valuable and sensitive tool for understanding the complicated pathological processes underlying RRMS. The considerable patient-dependent variability in VW-MTR changes in NABT and lesion volumes suggests that there may be heterogeneity in the population examined, which would merit further research in a larger and longer-term study to understand potential correlations between advanced MRI activity, disease activity and duration and relapses. In particular, it would be valuable to identify patients who would benefit most from MS therapies like IFN β-1a SC in terms of remyelination and whether the significant increase in VW-MTR signal suggestive of remyelinating activity in patients seen at 12 weeks can be sustained long-term.

## Supporting Information

Checklists S1 CONSORT Checklist.
(DOC)

Protocol S1 Trial Protocol.
(DOC)
