# Supplementary material for: Effect of Treatment with Interferon Beta-1a on Changes in Voxel-Wise Magnetization Transfer Ratio in Normal Appearing Brain Tissue and Lesions of Patients with Relapsing–Remitting Multiple Sclerosis: A 24-Week, Controlled Pilot Study
Source: PLoS One. 2014 Mar 13;9(3):e91098. doi: 10.1371/journal.pone.0091098 (PMC3953325; doi:10.1371/journal.pone.0091098)
Supplement: Protocol S1 — Trial Protocol. (DOC) [file pone.0091098.s002.doc]

# Clinical Trial Protocol

| **Clinical Trial Protocol Number** | 29655 |
| --- | --- |
| **Title** | A twenty-four week, two arm, pilot trial to evaluate remyelination/demyelination, gray matter volume and iron deposition in the central nervous system (CNS) and immune status of subjects with relapsing-remitting multiple sclerosis (RRMS) treated with Rebif® 44 mcg subcutaneously (sc) three times a week (tiw) compared to a healthy control group |
| **Trial Phase** | Pilot Trial |
| **Principal Study Investigator**  **Principal Site Investigator:**  **Immunologic Studies Principal Investigator:** | Robert Zivadinov, MD, PhD  Buffalo Neuroimaging Analysis Center  The Jacobs Neurological Institute  Department of Neurology  State University of New York at Buffalo  Buffalo, NY, USA  Bianca Weinstock-Guttman, MD  The Jacobs Neurological Institute  Department of Neurology  State University of New York at Buffalo  Buffalo, NY, USA  Silva Markovic-Plese, MD  Multiple Sclerosis Division  Department of Neurology  Chapel Hill, NC 27599-7025 |
| **Sponsor** | EMD Serono, Inc.  One Technology Place  Rockland, MA 02370  Ph: +1-781-982-9000  **Medical Responsible:**  Fernando Dangond, MD  Sr. Medical Director, Medical Affairs Neurology  EMD Serono, Inc.  One Technology Place, Rockland, MA 02370, USA  Ph: +1-781-681-2348  Fax: +1-781-681-2902 |
| **Clinical Trial Protocol Version** | Final 16 Dec 2009/Version 1.0 |

- Confidential -

This document is the property of Merck KGaA, Darmstadt, Germany, or one of its affiliated companies. It is intended for restricted use only and may not – in full or part – be passed on, reproduced, published or used without express permission of Merck KGaA, Darmstadt, Germany, or its affiliate. Copyright © 2*009* by Merck KGaA, Darmstadt, Germany, or its affiliate. All rights reserved.

**Signature Page**

**Protocol Lead responsible for designing the clinical trial:**

I approve the design of the clinical trial.

_____________________________________ ____________________________

Signature Date of Signature

Fernando Dangond, MD

Sr. Medical Director, Medical Affairs Neurology

EMD Serono, Inc.

One Technology Place, Rockland, MA 02370

Ph: +1-781-681-2348

Fax: +1-781-681-2902

fernando.dangond@emdserono.com

**Principal Study Investigator**

I agree to conduct the clinical trial in accordance with this clinical trial protocol and in compliance with Good Clinical Practice and all applicable regulatory requirements.

_____________________________________ ____________________________

Signature Date of Signature

Robert Zivadinov, MD, PhD

Buffalo Neuroimaging Analysis Center

The Jacobs Neurological Institute

Department of Neurology

State University of New York at Buffalo

Buffalo, NY, USA

100 High Street

Buffalo 14203 NY

Phone: 716-859-3579

Fax: 716-859-4005

Email: [rzivadinov@bnac.net](mailto:rzivadinov@bnac.net)

**Principal Site Investigator**

I agree to conduct the clinical trial in accordance with this clinical trial protocol and in compliance with Good Clinical Practice and all applicable regulatory requirements.

_____________________________________ ____________________________

Signature Date of Signature

Bianca Weinstock-Guttman, MD

The Jacobs Neurological Institute

Department of Neurology

State University of New York at Buffalo

Buffalo, NY, USA

**Principal Investigator – Immunological Studies**

I agree to conduct the clinical trial in accordance with this clinical trial protocol and in compliance with Good Clinical Practice and all applicable regulatory requirements.

_____________________________________ ____________________________

Signature Date of Signature

Silva Markovic-Plese, MD

Multiple Sclerosis Division

Department of Neurology

Chapel Hill, NC 27599-7025

**Further Sponsor Responsible Persons**

Other individuals from the Sponsor’s organization contributing to this Protocol:

| Contributor and Contact Information | Contributor and Contact Information |
| --- | --- |
| Maryellen Craig  Project Manager  Medical Operations  EMD Serono, Inc.  One Technology Place  Rockland, MA 02370  Phone: 781-681-2223  Fax: 781-681-2902 | Brooke Hayward, SM, MBA  Sr. Principal Biostatistician  Medical Operations  EMD Serono, Inc.  One Technology Place  Rockland, MA 02370  Phone: 781-681-2366  Fax: 781-681-2902 |
| Lisa Hyde, RAC  Specialist, Global Regulatory Affairs  Neurodegenerative Diseases  EMD Serono, Inc.  One Technology Place  Rockland, MA 02370  Phone: 781-681-2356  Fax: 781-681-2924 | Yuhong Yang, RN  Drug Safety Officer,  Global Drug Safety  EMD Serono, Inc.  One Technology Place  Rockland, MA 02370  Phone: 781-681-2839  Fax: 781-681-2915 |

**Principal Investigator Signature**

**Trial Title** A twenty-four week, two arm, pilot trial to evaluate remyelination/demyelination, gray matter volume and iron deposition in the central nervous system (CNS) and immune status of subjects with relapsing-remitting multiple sclerosis (RRMS) treated with Rebif® 44mcg subcutaneously (sc) three times a week (tiw) compared to a healthy control group

**Clinical Trial Protocol Version/Date** Final 16 Dec 2009/Version 1.0

**Center Number**

**Principal Investigator**

I, the undersigned, am responsible for the conduct of the trial at this site and affirm that:

- I understand and will conduct the trial according to the clinical trial protocol, any approved protocol amendments, ICH Good Clinical Practice (ICH Topic E6 GCP) and all applicable Health Authority requirements and national laws.
- I will not deviate from the clinical trial protocol without prior written permission from the Sponsor and prior review and written approval from the Institutional Review Board, except where necessary to prevent immediate danger to the subject.

I understand that some Health Authorities require the Sponsors of clinical trials to obtain and supply, when required, details about the Investigators’ ownership interests in the Sponsor or Medicinal Product and information regarding any financial ties with the Sponsor. The Sponsor will use any such information that is collected solely for the purpose of complying with the regulatory requirements. I therefore agree to supply the Sponsor with any necessary information regarding ownership interest and financial ties (including those of my spouse and dependent children), and to provide updates as necessary.

_____________________________________ ____________________________

Signature Date of Signature

Name, academic degree

Function

Institution

Address

Telephone number

Fax number

E-mail address

# Table of Contents

[1 Synopsis 16](#__RefHeading___Toc248727612)

[2 Sponsor, Investigators and Trial Administrative Structure 20](#__RefHeading___Toc248727613)

[3 Background Information 21](#__RefHeading___Toc248727614)

[3.1 Description of MRI Techniques 21](#__RefHeading___Toc248727615)

[3.2 Description of Medicinal Product 24](#__RefHeading___Toc248727616)

[3.3 Rebif® (Interferon beta-1a) 24](#__RefHeading___Toc248727617)

[3.4 Rationale for Current Trial 25](#__RefHeading___Toc248727618)

[3.5 Summary of Overall Risks and Benefits 26](#__RefHeading___Toc248727619)

[3.6 Background of MS 26](#__RefHeading___Toc248727620)

[3.7 Clinical Variants of MS 27](#__RefHeading___Toc248727621)

[3.8 Summary of Rebif® Pre-Clinical Trials 27](#__RefHeading___Toc248727622)

[3.9 Summary of Rebif® Clinical Trials 27](#__RefHeading___Toc248727623)

[3.10 Safety Profile of Rebif® based on Clinical Trials 28](#__RefHeading___Toc248727624)

[4 Trial Objectives 29](#__RefHeading___Toc248727625)

[4.1 Primary Objective 29](#__RefHeading___Toc248727626)

[4.2 Secondary Objectives 29](#__RefHeading___Toc248727627)

[5 Investigational Plan 30](#__RefHeading___Toc248727628)

[5.1 Overall Trial Design and Plan 30](#__RefHeading___Toc248727629)

[5.1.1 Study Design 30](#__RefHeading___Toc248727630)

[5.1.2 Primary and Secondary Endpoints 30](#__RefHeading___Toc248727631)

[5.1.3 Safety Endpoints 31](#__RefHeading___Toc248727632)

[5.1.4 Study Subjects 31](#__RefHeading___Toc248727633)

[5.1.5 Study Treatment with Medicinal Product 31](#__RefHeading___Toc248727634)

[5.1.6 Blinding and Randomization 32](#__RefHeading___Toc248727635)

[5.1.7 Overall Duration of the Study 32](#__RefHeading___Toc248727636)

[5.1.8 Schedule of Assessments 32](#__RefHeading___Toc248727637)

[5.2 Discussion of Trial Design 34](#__RefHeading___Toc248727638)

[5.3 Inclusion of Special Populations 34](#__RefHeading___Toc248727639)

[5.4 Selection of Trial Population 35](#__RefHeading___Toc248727640)

[5.4.1 Inclusion Criteria 35](#__RefHeading___Toc248727641)

[5.4.2 Exclusion Criteria 36](#__RefHeading___Toc248727642)

[5.4.3 Exceptions to the Eligibility Criteria 37](#__RefHeading___Toc248727643)

[5.4.4 Criteria for Subject Withdrawal 37](#__RefHeading___Toc248727644)

[5.4.5 Withdrawal from the Trial 37](#__RefHeading___Toc248727645)

[5.4.6 Replacement of Discontinued Subjects 38](#__RefHeading___Toc248727646)

[5.4.7 Follow-up for Discontinued Subjects 38](#__RefHeading___Toc248727647)

[5.4.8 Premature Discontinuation of the Trial 39](#__RefHeading___Toc248727648)

[5.4.9 Definition of End of Trial 39](#__RefHeading___Toc248727649)

[5.4.10 Medical Care of Subjects after End of Trial 39](#__RefHeading___Toc248727650)

[6 Medicinal Product(s) and Other Drugs Used in the Trial 39](#__RefHeading___Toc248727651)

[6.1 Dosage and Administration 39](#__RefHeading___Toc248727652)

[6.2 Packaging and Labeling 40](#__RefHeading___Toc248727653)

[6.3 Preparation, Handling and Storage 40](#__RefHeading___Toc248727654)

[6.4 Concomitant Medications and Therapies 40](#__RefHeading___Toc248727655)

[6.4.1 Permitted Medicines 40](#__RefHeading___Toc248727656)

[6.4.2 Non-permitted Medicines 41](#__RefHeading___Toc248727657)

[6.4.3 Concomitant Procedures 41](#__RefHeading___Toc248727658)

[6.4.4 Special Precautions 41](#__RefHeading___Toc248727659)

[6.5 Medicinal Product Accountability 42](#__RefHeading___Toc248727660)

[6.6 Assessment of Medicinal Product Compliance 42](#__RefHeading___Toc248727661)

[6.7 Method of Blinding 43](#__RefHeading___Toc248727662)

[6.8 Emergency Unblinding 43](#__RefHeading___Toc248727663)

[6.9 Treatment of Overdose 43](#__RefHeading___Toc248727664)

[6.10 Medical Care of Subjects after End of Trial 43](#__RefHeading___Toc248727665)

[7 Trial Procedures and Assessments 43](#__RefHeading___Toc248727666)

[7.1 Schedule of Assessments 43](#__RefHeading___Toc248727667)

[7.2 Assessments to be performed at each scheduled visit 44](#__RefHeading___Toc248727668)

[7.2.1 Screening Period 44](#__RefHeading___Toc248727669)

[7.2.2 Treatment Period 45](#__RefHeading___Toc248727670)

[7.2.3 Post-Treatment Period 48](#__RefHeading___Toc248727671)

[7.2.4 Laboratory Assessments 48](#__RefHeading___Toc248727672)

[7.2.5 Immunological Assessments 48](#__RefHeading___Toc248727673)

[7.2.6 Other Assessments 49](#__RefHeading___Toc248727674)

[7.3 Pharmacokinetics 52](#__RefHeading___Toc248727675)

[7.4 Body Fluid(s) 53](#__RefHeading___Toc248727676)

[7.5 Pharmacokinetic Calculations 53](#__RefHeading___Toc248727677)

[7.6 Biomarkers/Pharmacogenetics (PGx) 53](#__RefHeading___Toc248727678)

[7.7 Other Assessments 53](#__RefHeading___Toc248727679)

[7.8 Assessment of Efficacy 53](#__RefHeading___Toc248727680)

[7.9 Assessment of Safety 53](#__RefHeading___Toc248727681)

[7.9.1 Adverse Events 54](#__RefHeading___Toc248727682)

[7.9.2 Pregnancy and In Utero Drug Exposure 60](#__RefHeading___Toc248727683)

[8 Statistics 61](#__RefHeading___Toc248727684)

[8.1 Sample Size 61](#__RefHeading___Toc248727685)

[8.2 Randomization 61](#__RefHeading___Toc248727686)

[8.3 Analysis Sets 61](#__RefHeading___Toc248727687)

[8.4 Description of Statistical Analyses 61](#__RefHeading___Toc248727688)

[8.4.1 General Considerations 61](#__RefHeading___Toc248727689)

[8.4.2 Analysis of Primary Endpoint(s) 62](#__RefHeading___Toc248727690)

[8.4.3 Analysis of Secondary Endpoint(s) 62](#__RefHeading___Toc248727691)

[8.4.4 Safety Analyses 62](#__RefHeading___Toc248727692)

[8.4.5 Analysis of Further Endpoints 63](#__RefHeading___Toc248727693)

[8.5 Interim Analysis 63](#__RefHeading___Toc248727694)

[9 Ethical and Regulatory Aspects 63](#__RefHeading___Toc248727695)

[9.1 Responsibilities of the Investigator 63](#__RefHeading___Toc248727696)

[9.2 Subject Information and Informed Consent 63](#__RefHeading___Toc248727697)

[9.3 Subject Identification and Privacy 64](#__RefHeading___Toc248727698)

[9.4 Emergency Medical Support and Subject Card 64](#__RefHeading___Toc248727699)

[9.5 Institutional Review Board 65](#__RefHeading___Toc248727700)

[9.6 Health Authorities 65](#__RefHeading___Toc248727701)

[10 Trial Management 65](#__RefHeading___Toc248727702)

[10.1 Case Report Form Handling 65](#__RefHeading___Toc248727703)

[10.2 Source Data and Subject Files 66](#__RefHeading___Toc248727704)

[10.2.1 Hospitalization Records 67](#__RefHeading___Toc248727705)

[10.3 Investigator Site File and Archiving 67](#__RefHeading___Toc248727706)

[10.4 Monitoring, Quality Assurance and Inspection by Health Authorities 67](#__RefHeading___Toc248727707)

[10.5 Changes to the Clinical Trial Protocol 68](#__RefHeading___Toc248727708)

[10.6 Clinical Trial Report and Publication Policy 68](#__RefHeading___Toc248727709)

[10.6.1 Clinical Trial Report 68](#__RefHeading___Toc248727710)

[10.6.2 Publication 68](#__RefHeading___Toc248727711)

[11 References 69](#__RefHeading___Toc248727712)

[12 Appendices 75](#__RefHeading___Toc248727713)

[12.1 Appendix A - Schedule of Assessments 76](#__RefHeading___Toc248727714)

[12.2 Appendix B - Laboratory Parameters 78](#__RefHeading___Toc248727715)

[12.3 Appendix C- Common Terminology Criteria for Adverse Events 79](#__RefHeading___Toc248727716)

**List of Abbreviations**

| AE | Adverse Event |
| --- | --- |
| ALT | Alanine Aminotransferase |
| ANCOVA | Analysis of Covariance |
| BDNF | Brain Derived Neurotrophic Factor |
| CD | Cluster of Differentiation |
| CFR | Code of Federal Regulations |
| CNS | Central Nervous System |
| CSF | Cerebral Spinal Fluid |
| CTCAE | Common Terminology Criteria for Adverse Events |
| DGM | Deep Gray Matter |
| e-CRF | Electronic Case Report Form |
| EDSS | Expanded Disability Status Scores |
| FDA | Food and Drug Administration |
| FLAIR | Fluid Attenuated Inversion Recovery |
| FLIRT | FMRIB’s Linear Image Registration Tool |
| FLS | Flu-Like Symptoms |
| GCP | Good Clinical Practice |
| Gd | Gadolinium |
| GM | Gray Matter |
| GRE | Gradient Echo |
| HR | Hazard Ratio |
| HIPAA | Health Insurance Portability and Accountability Act |
| HC | Healthy Controls |
| hCG | Human Chorionic Gonadotropin |
| ICF | Informed Consent Form |
| ICH | International Conference on Harmonization |
| IFN-beta | Interferon beta |
| IRB | Institutional Review Board |
| IR-FSPGR | Inversion Recovery Fast-Spoiled Gradient Recalled |
| ISR(s) | Injection Site Reaction(s) |
| ITT | Intent to Treat |
| LV | Lesion Volume |
| minIP | Minimum Intensity Projection |
| mL | Milliliters |
| MR | Magnetic Resonance |
| MRI | Magnetic Resonance Imaging |
| MS | Multiple Sclerosis |
| MTI | Magnetization Transfer Imaging |
| MTR | Magnetization Transfer Ratio |
| NABT | Normal Appearing Brain Tissue |
| NAGM | Normal Appearing Gray Matter |
| NAWM | Normal Appearing White Matter |
| NGF | Nerve Growth Factor |
| PDF | Portable Document Format |
| PGx | Pharmacogenetics/Pharmacogenomics |
| PPMS | Primary Progressive Multiple Sclerosis |
| PRMS | Progressive-Relapsing Multiple Sclerosis |
| ROI | Region of Interest |
| ROS | Reactive Oxygen Species |
| RRMS | Relapsing-Remitting Multiple Sclerosis |
| SAE | Serious Adverse Event |
| sc | Subcutaneous |
| SE | Spin-Echo |
| SIENAX | Structural Image Evaluation, using Normalization, of Atrophy, Cross-Sectional |
| SPMS | Secondary Progressive Multiple Sclerosis |
| SUSAR | Suspected Unexpected Serious Adverse Reaction |
| SWI | Susceptibility-Weighted Imaging |
| TFCE | Threshold-Free Cluster Enhancement |
| tiw | Three Times a Week |
| TSH | Thyroid Stimulating Hormone |
| ULN | Upper Limit of Normal |
| VW | Voxel-Wise |
| VW-MTR | Voxel-Wise Magnetization Transfer Ratio |
| WI | Weighted Image |
| WM | White Matter |

# Synopsis

| **Trial title** | A Twenty-Four week, two arm, pilot trial to evaluate remyelination/demyelination, gray matter volume and iron deposition in the central nervous system (CNS) and immune status of subjects with relapsing-remitting multiple sclerosis (RRMS) treated with Rebif® 44mcg subcutaneously (sc) three times a week (tiw) |
| --- | --- |
| **Trial number** | 29655 |
| **EudraCT number** | N/A |
| **Sponsor** | EMD Serono, Inc. |
| **Phase** | Pilot Trial |
| **Trial under IND** | yes  no |
| **FDA "covered trial"** | yes  no |
| **Trial center(s)/country(ies)** | One US Center |
| **Planned trial period  (first enrollment-last subject out)** | Enrollment of twenty-five RRMS subjects and fifteen healthy control (HC) subjects is expected to commence February 2010 and continue until the enrollment goal is achieved. |
| **Trial objectives** | The primary objective is to characterize the effect of Rebif® 44 mcg sc tiw on remyelination/demyelination using a voxel-wise magnetization transfer ratio (VW-MTR) dynamic mapping of normal appearing brain tissue (NABT) in subjects with RRMS over six months of treatment, and with reference to a group of healthy controls (HC).  Secondary objectives are to characterize the effect of Rebif® 44 mcg on:   - remyelination/demyelination using a VW-MTR dynamic mapping of lesions (T2, T1 & Gd) in RRMS subjects over six months of treatment, and with reference to HC - brain volume using 3D-T1 imaging of whole brain matter in RRMS subjects over six months of treatment, and with reference to HC. - CNS iron deposition using Susceptibility-Weighted Imaging (SWI) of the deep gray matter (DGM), NABT and of the lesions in RRMS subjects over six months of treatment, and with reference to HC. - Lesion volume (LV) using T2-LV, T1-LV and T1-Gd-LV imaging in RRMS subjects over six months of treatment.   Further secondary objectives are to assess the following:   - Immune phenotype of T cells and monocytes, with special focus on the levels of cytokines involved in the pathogenic Th17 pathway - Immune cell production of neurotrophic factors, including nerve growth factor (NGF) and brain-derived neurotrophic factor (BDNF), in order to assess potential associations with remyelination, neurodegeneration, iron deposition or GM volumes - Ferritin and transferrin level assessment, in order to assess the relationship of CNS iron deposition with ferritin levels and with transferrin levels - Clinical relapses - Safety evaluation |
| **Trial design and plan** | This is a twenty-four week, two arm pilot trial of RRMS subjects compared to a healthy control reference group. A total of twenty-five eligible RRMS subjects will be enrolled, with scheduled treatment duration of twenty-four weeks plus a four-week, post-treatment telephone call. For treatment, subjects will receive Rebif® 44 mcg sc tiw. In addition, 15 HC will be enrolled. Baseline assessments include physical and neurological exams, magnetic resonance imaging (MRI) scans, laboratory tests (hematology, chemistry, urinalysis, ferritin, and blood samples for immunologic studies) and pregnancy testing. |
| **Planned number of subjects** | It is estimated that fifty -five subjects will be screened to achieve twenty-five intent to treat (ITT) RRMS subjects who will start therapy with Rebif® 44 mcg sc tiw and fifteen healthy controls. The fifteen HC subjects will be enrolled as a reference group according to the same age range requirements and the same ratio of males to females as the RRMS subjects. |
| **Schedule of visits and assessments** | Eligible subjects will undergo a screening assessment within fourteen days prior to entering the study. Thereafter, eligible subjects will return to the clinic for assessments on Study Day 1, Week 12 and Week 24/Exit. A safety evaluation telephone call will take place four weeks post-Exit visit to monitor the subjects’ status and well-being. |
| **Diagnosis and main inclusion and exclusion criteria** | Inclusion Criteria: RRMS participants who have been diagnosed according to the McDonald criteria, and have a disease duration of less than ten years. Subjects will consist of male and female subjects between 18-65 years of age. |
| **Medicinal Product (s): dose/mode of administration/ dosing schedule** | The term Medicinal Product refers to Rebif® 44 mcg for sc injection.  Injections should be administered sc tiw for twenty-four weeks. |
| **Reference therapy(ies): dose/mode of administration/ dosing schedule** | Not applicable. |
| **Planned treatment duration per subject** | The dosing regimen complies with the product labeling of the currently marketed formulation of Rebif®. Each single-administration, pre-filled syringe should be administered sc tiw over the twenty-four week treatment period. |
| **Primary endpoint(s)** | The primary endpoints are the numbers of voxels (counted in milliliters) that change in VW-MTR value (both increase and decrease) from baseline to six-months in NABT of RRMS subjects treated with Rebif® as compared to NABT of HC. |
| **Secondary endpoint(s)** | The secondary MRI endpoints for RRMS subjects treated with Rebif® are:   - the number of voxels that increase in VW-MTR value at three months vs. baseline in NABT - the increase and decrease in number of voxels from VW-MTR at three- and six-months vs. baseline in lesions - brain volume using 3D-T1 imaging at three- and six-months vs. baseline in whole brain and GM - the amount of iron deposition identified via SWI at three- and six-months vs. baseline in DGM, NABT and lesions - lesion volume from T2-LV, T1-LV and T1-Gd-LV imaging at three- and six-months vs. baseline   Other secondary endpoints of the study examine potential associations of the effects of Rebif® in the CNS with peripheral effects on:   - number of clinical relapses - time to first clinical relapse - immune cell phenotype: related to CNS infiltration (Th17 cells) - immune cell production of neurotrophic factors: related to remyelination or preservation of gray matter volume, and - ferritin and transferrin levels: potentially associated with levels of CNS iron deposition.   The safety endpoints are to determine the incidence of adverse events following administration of study treatment with Rebif® over six months via electronic case report form (e-CRF) based adverse event collection. |
| **Pharmacokinetics** | Not Applicable |
| **Other assessments** | Not Applicable |
| **Statistical methods (includes sample size calculation)** | Sample size is based on clinical rather than statistical considerations.  All statistical tests will be two-tailed and considered significant at the α=0.05 level of significance.  T-tests and chi-square tests for independent samples will be used for comparison of demographic and clinical characteristics between RRMS patients and HC. For testing differences across groups, an analysis of covariance (ANCOVA) on the ranked data with effects for treatment group (RRMS vs. HC), treatment period (1^st^ 3 months vs. 2^nd^ 3 months), age, and gender will be performed. Model assumptions of linearity, normality and homoschedasticity will be verified. For testing differences across time (at week 12 and 24) within the RRMS treatment group, the Wilcoxon signed rank test will be used.  VW-MTR spatial distribution analysis will be conducted directly on the images. Similarly, voxel-wise SWI spatial distribution analysis will be conducted directly on the images.  Analysis of MRI measures may require a variance stabilizing transformation, if skewness is observed in their assessments. Each of the MRI parameters will be examined and appropriate transformations will be applied, if necessary. |

# Sponsor, Investigators and Trial Administrative Structure

A description of the administrative structure of this trial is as follows:

**Trial Sponsor**: The Sponsor of the trial is EMD Serono, Inc., Rockland, MA, 02370, USA. Rebif® is manufactured by EMD Serono, Inc. and co-marketed by EMD Serono, Inc. and Pfizer, Inc., 235 E. 42^nd^ Street, New York, NY 10017, USA.

**Location(s) of the Trial**: This single center trial is to be conducted in the United States.

**Principal Investigator:** The Principal Investigator will serve as the representative for decisions and discussions regarding this trial (ICH E6 GCP, 1.19). His role will be to provide expert medical input and advice relating to the trial design and conduct, and he will be responsible for the review and sign-off of the Clinical Trial Report. The Principal Investigator’s contact information is located on pages 1 and 2 of this protocol.

**Buffalo Neuroimaging Center (BNAC):** BNAC will be performing image analysis on all MRIs.

**Jacobs Neurological Institute** will serve as the clinical site responsible for recruiting all subjects, obtain laboratory samples, performing clinical assessments and distributing Medicinal Product.

**University of North Carolina – Chapel** **Hill** will provide analysis for immunological samples.

In compliance with Food and Drug Administration (FDA) regulations, the Investigator will submit this protocol to a certified institutional review board (IRB) at the Jacobs Neurological Institute for review and approval prior to study initiation at the study site. **Independent Oversight Committees or Boards**: Since Rebif® is commercially available and the safety profile is known and understood, it is not considered necessary to convene a Data Monitoring Committee, a Safety Monitoring Board, or a Steering Committee to provide oversight on the safety of Rebif® during the conduct of this trial. That responsibility will be assumed by the Sponsor’s Drug Safety department (see “Global Drug Safety Responsibilities” below).

**Responsibilities for Drug Supply and Distribution**: The Sponsor will rely on qualified, regional vendors to distribute shipments of commercial Rebif® and other clinical supplies directly to the study site.

**Global Drug Safety Responsibilities**: The Sponsor’s Drug Safety department will be responsible for overseeing the safety of Rebif® during the conduct of this trial and will inform the participating study site and the FDA of any serious or other reportable adverse events or untoward safety signals.

# Background Information

This clinical trial will be conducted in compliance with the clinical trial protocol, Good Clinical Practice (ICH Topic E6, GCP) and the applicable regulatory requirements.

## Description of MRI Techniques

Multiple sclerosis (MS) is a chronic, immune-mediated disease of the central nervous system (CNS). It is a complex, multi-factorial disease that includes inflammatory and neurodegenerative processes manifesting both focally in the form of lesions and diffusely in otherwise normal-appearing brain tissue (NABT) (1)). The key hallmarks of this disease are the ongoing processes of demyelination and remyelination (2-3). Studies using magnetic resonance imaging (MRI) (2)(4)(5), histopathological analysis (2,6-8), and animal models (9) have demonstrated that these processes occur in lesions as well as in NABT. The ability to detect both demyelination and remyelination in vivo could provide substantial benefits, including better tracking of individual patient responses to therapy and better overall understanding of the effects of various therapies on disease progression.

Magnetization transfer ratio (MTR) analysis takes advantage of this phenomenon by measuring and quantifying the degree of signal loss at each voxel. Post-mortem and animal model histopathological investigations have confirmed that this approach is very sensitive to tissue myelin content, and is capable of detecting both demyelination and remyelination (2,6,10-12). Given its sensitivity for myelin content, longitudinal application of MTR is a natural candidate for tracking demyelination and remyelination in vivo.

Despite its potential, however, analysis techniques have been limited in extracting this information from longitudinal MTR images. To date, most have been histogram-based approaches looking at measures of central tendency (generally mean or median MTR) in a priori regions of interest (ROIs), such as tissue segmentation maps or hand-drawn anatomical masks (5,13-21). Although these techniques can provide a great deal of information, they are limited in two important respects. First, they rely on a priori location assumptions, and may therefore be less sensitive to changes that do not occur exactly within predefined boundaries. Second, and perhaps most important, they are sensitive only to mean change in the area being analyzed rather than the overall level of activity; competing processes of demyelination and remyelination may cancel each other out and result in a measurement falsely suggesting a lack of disease activity.

More recent techniques (6,10-11) have used a voxel-wise (VW) approach to MTR change measurement that addresses the shortcomings of ROI-histogram approaches. By looking at each voxel independently and assessing direction and degree of change, they avoid the need for a priori ROIs. By adding a threshold for activity, they are able to avoid the histogram-mean-related problem of opposite processes canceling out. Specific techniques proposed have been either group-based, similar in concept to voxel-based-morphometry (22), or single-subject-based (6).

The group-based approach provides a high degree of statistical rigor, but its utility is greatly restricted by its reliance on the unlikely assumption that areas of change will be in close proximity in all subjects. The single-subject approach removes this constraint, but at the cost of greatly reducing sensitivity. With only a single observation per voxel, random noise in the underlying MTR maps results in the need for a very high change threshold, making it easy to miss large areas of subtle change.

To address the remaining unsolved MTR analysis issues and potentially improve the ability to detect demyelination and remyelination in vivo, a sensitive, noise-resistant technique for single-subject VW longitudinal MTR change analysis by combining threshold-free cluster enhancement (TFCE) with a robust M-estimator technique and a Monte Carlo approach (23) has been used. TFCE is first applied to enhance individual voxels based on their level of local cluster support, and then Monte Carlo estimation is performed to allow meaningful statistical interpretation of the resulting TFCE values. The technique was validated in three complementary ways: healthy control scan-rescan analysis, analysis of a “gold standard” simulated dataset, and analysis of a group of MS patients and healthy volunteers with 1-year longitudinal MRI scans.

Scan-rescan analysis has demonstrated a very low false-positive rate (1.44mL increasing and 1.48mL decreasing at the optimal detection threshold). Simulated dataset analysis has yielded an area under receiver-operating characteristic curve of 0.942 (compared to 0.801 for a more conventional VW thresholding analysis). Finally, analysis of the real subject population has shown highly significant differences (p<0.001) in volume of decreasing MTR between patients and controls. The proposed method provides a valuable means for quantifying MS-related tissue changes, particularly demyelination and remyelination, in vivo and without the use of highly complex or experimental MRI acquisition techniques. It improves on the sensitivity of other approaches, and may increase the statistical power of studies investigating the effects of therapy on MRI outcomes in MS.

Given its demonstrated sensitivity, specificity and reproducibility, the VW-MTR approach has a number of potential applications. In its most straightforward interpretation, the proposed method provides increased ability to detect competing processes of demyelination and remyelination in vivo, and therefore the ability to better understand the dynamic nature of MS disease processes.

In addition, tracking VW-MTR changes may provide a means for synthesizing information about a variety of tissue changes not individually detectable with any other single MRI modality. Several strategies were proposed over the last decade to overcome the limitations of conventional MRI scanning for better visualization of the overall MS pathology. More advanced sequences (double inversion recovery and 3D-FLAIR) (24-25), as well as new contrast agents (26) are able to detect in vivo gray matter (GM) and white matter (WM) pathology that is currently invisible with standard conventional sequences. Unfortunately, current MRI protocols (including high and ultra-high-field imaging) are not able to detect lesions in GM (type III lesions). These lesions are now known to be widespread in subpial regions (27-28), and may be a critical factor in the development of irreversible disability.

The advantage of the VW-MTR method is its ability to capture the above pathological changes in a single approach, independent of the underlying inflammatory or neurodegenerative processes. In fact, others have demonstrated that MTR changes can be used to identify cortical lesions not visible with more conventional MTI techniques (29), and our current data confirms that our proposed method is capable of taking advantage of MTR's sensitivity, both in normal appearing gray matter (NAGM) and normal appearing white matter (NAWM).

Susceptibility Weighted Imaging (SWI) is a new neuroimaging technique, which uses tissue magnetic susceptibility differences to generate a unique contrast, different from that of spin density, T1, T2, and T2* (30-31). SWI consists of using both magnitude and phase images from a high-resolution, three-dimensional fully velocity-compensated gradient echo sequence. Phase masks are created from the MR phase images, and multiplying these with the magnitude images increases the conspicuousness of the smaller veins and other sources of susceptibility effects, which is depicted using minimum intensity projection (minIP). The phase images are useful in differentiating between diamagnetic and paramagnetic susceptibility effects of calcium and blood, respectively. This unique MR sequence will help in detecting occult low flow vascular lesions, calcification and cerebral microbleeds in various pathologic conditions and aids in characterizing tumors and degenerative diseases of the brain. This sequence can also be used for the prominent display of normal brain structures. Using phase as an iron marker may be useful for studying absorption of iron in diseases such as Parkinson's, Huntington's, neurodegeneration with brain iron accumulation, Alzheimer's, and MS and other iron-related diseases.

The use of SWI in MS has gained increasing attention recently. Although, the use of SWI in MS is in its infancy, the detection of vein abnormalities and areas of iron deposition in the brain are two main topics of interest (32). Some have demonstrated markedly enhanced detection of unique microvascular involvement associated with most of the visualized MS lesions with abnormal signals on and around the venous wall on 7-T compared with 3-T MRI. These findings, which have never been seen on conventional fields of MRI, allowed for direct evidence of vascular pathogenesis in MS in vivo and have important implications for monitoring lesion activity and therapeutic response. Hammond et al (33) studied seven patients with MS and observed small phase-shifted plaques in the region of the deep medullary veins; this observation supports the contention that the disease progresses along the vasculature. They found that a subset of MS plaques showed contrast uptake only at the peripheral margin of the lesion in the phase images. More recently, (30) completed a study of twenty-seven patients with MS by using SWI filtered phase images. They found that there is a negative correlation of iron with T2 hyperintensity. The higher the iron content, the less visible the lesions seem to be on T2-weighted images (WI). This suggests that significant damage to the tissue occurs following chronic inflammation, such that the tissue no longer shows signs of inflammation. They also noted that there were marked hypointense rings around some lesions, lesions with uniform iron content, and regions with central iron but no rings. On occasion, lesions were also seen in the GM as well. Overall, in that particular study, using SWI permitted a more accurate measurement of the extent of MS and increased the number of lesions detected by 50% in the WM and GM (30).

## Description of Medicinal Product

Refer to the Rebif® Package Insert for specific information regarding the currently marketed formulation of Interferon-beta-1a.

In this trial, the term Medicinal Product refers to Rebif® 44 mcg in a 0.5 mL pre-filled syringe for subcutaneous (sc) injection [(see Section 6)](#_Dosage_and_Administration).

## Rebif® (Interferon beta-1a)

Rebif® (interferon beta-1a) is a purified 166 amino acid glycoprotein with a molecular weight of approximately 22,500 daltons. It is produced by recombinant DNA technology using genetically engineered Chinese Hamster Ovary cells into which the human interferon beta gene has been introduced. The amino acid sequence of Rebif® is identical to that of natural fibroblast derived human interferon beta. Natural interferon beta and interferon beta-1a (Rebif®) are glycosylated with each containing a single N-linked complex carbohydrate moiety.

Rebif® has been tested in a series of studies in MS subjects at doses ranging from 22 mcg to 132 mcg weekly with a dose frequency ranging from weekly to thrice weekly. The pivotal trial in RRMS was the **P**revention of **R**elapses and Disability by **I**nterferon beta-1a **S**ubcutaneously in **M**ultiple **S**clerosis (PRISMS) study (34-35), a double-blind, multicenter placebo controlled randomized trial of 22 mcg tiw or 44 mcg tiw. The initial two-year study demonstrated that Rebif® at both doses significantly reduced relapse rate (32% reduction for 44 mcg tiw relative to placebo, p<0.001), slowed disability progression (Hazard Ratio, [HR]=0.63, p=0.01) and reduced MRI lesion activity (77% reduction T2 active lesions, p<0.0001) and accumulation of new lesion burden (-6.6% compared to +11%, p<0.0001). Rebif® is the only disease modifying agent to have demonstrated efficacy on all four of these efficacy measures in RRMS.

## Rationale for Current Trial

The purpose of this trial is to evaluate the effects of Rebif® 44 mcg subcutaneous (sc) three times a week (tiw) on a) remyelination/demyelination, b) lesion and brain volume, c) CNS iron deposition, and d) immune status in subjects with RRMS via several MRI techniques.

It has been shown in several phase II, III and IV clinical trials that Rebif® 44 mcg had a robust effect on the decrease of inflammatory MRI activity and clinical activity as measured by reduction of clinical relapses and slowing in progression of disability. The potential effect of Rebif® 44 mcg on remyelination is unknown. This study will address these important questions by using voxel-wise MTR dynamic mapping of lesions and NABT to examine the evolution of these measures over six months.

Iron is essential for cellular physiology and normal metabolism of the body. It is required by all living cells due to its role in tissue formation, angiogenesis, oxygen transport, electron transfer, neurotransmitter synthesis, and myelin production; however, excess iron can be harmful (36). In excess, it contributes to a number of neurodegenerative processes because free ferrous (Fe++) iron reacts with peroxides to produce free radicals, which are highly reactive and can damage DNA, proteins, lipids, and other cellular components. In that case, both free and bound iron lead to toxic cellular effects. Free iron generally occurs when iron levels exceed the capacity of transferrin to bind the iron. Transferrin is a glycoprotein that is responsible for iron homeostasis in vivo and maintains iron pool in the systemic circulation by binding with iron tightly but reversibly. Free circulating iron, (contrary to ferritin-bound stable iron when converted to oxyhydroxyl derivatives and hemosiderin) may initiate and propagate the production of free radicals, leading to lipid peroxidation. Moreover, oxidative stress arises due to a disturbed equilibrium between pro-oxidant/antioxidant homeostasis that further takes part in generation of Reactive Oxygen Species (ROS) and free radicals with potential toxicity to neuronal cells. The reason for neuronal cell hypersensitivity towards oxidative stress arises due to anatomic and metabolic factors. In the brain, various types of glial cells are present and these are involved in anatomic support and metabolic requirement (37). The endothelial cells surrounding these glial cells are less permeable for uptake of various molecules. In addition, glial cells in brain require more oxygen and glucose consumption to generate continuous ATP pool in vivo for normal functioning of brain with enhanced susceptibility to free radical injury.

The excessive amount of iron detected in MS patients by a variety of different MRI techniques, including SWI, and therapeutic strategies against iron accumulation in MS are becoming highly important research topics in this field.

Therefore, measurement of iron accumulation in the white matter (WM) and particularly in deep gray matter (DGM) of patients with MS may become a very important outcome for future clinical trials and targets of therapeutic intervention.

Although the effect of Rebif® 44 mcg on iron accumulation and iron mediated damage in MS patients is unknown, given its mechanism of action and its potent effect on the blood-brain-barrier breakdown recovery, it is an ideal drug candidate to test whether it can slow down iron accumulation in the WM and DGM in patients with MS.

The study will provide the following:

- An understanding of the effects of Rebif® on previously unexplored mechanisms (i.e., remyelination and iron deposition in the CNS)
- Elucidation of potential associations of above results with effects of Rebif® on pathogenic (T helper 17, Th17) immune cells
- Examine the association of above results with the ability of immune cells to produce neurotrophic factors
- Inform the design of future clinical trials that aim to incorporate these advanced technologies to investigate treatment outcomes
- Advance the science and medicine of Rebif®.

## Summary of Overall Risks and Benefits

Subjects will receive Rebif® at no cost for the duration of the trial. The use of Rebif® may not directly benefit subjects during the trial.

Common adverse events that occur in Rebif® treated patients include injection site reactions, injection site pain, flu-like symptoms, hepatic dysfunction and cytopenia. Skin rash, depressive symptoms and thyroid dysfunction have also been reported with Rebif® [(See Section 3.10)](#_Safety_Profile_of)

## Background of MS

Multiple sclerosis (MS) is a chronic, inflammatory, demyelinating disease of the central nervous system and is the most common cause of neurological disability in young adults. It is characterized by multi-focal recurrent attacks of neurological symptoms and signs with variable recovery. Eventually, the majority of patients develop a progressive clinical course (38-40)(39-41).

The exact cause of MS is unknown, although an autoimmune process has been implicated.

Genetic susceptibility plays a role in disease initiation, (42-43) (43) but currently unidentified environmental factors are also likely involved (44)

It is hypothesized that central nervous system (CNS) auto-reactive T cells are stimulated in the peripheral circulation to become active and proliferate. The expression of adhesion molecules on the surface of T cells permits adhesion to activated endothelial cells with subsequent migration of T cells into the CNS compartment. These cells upon interacting with CNS myelin antigens again proliferate, and initiate a pro-inflammatory cascade within the brain that results in either target-directed immune damage or bystander damage. Important cellular and humoral elements are T-lymphocytes, B-lymphocytes, dendritic cells, macrophages, microglial cells, metalloproteinases and pro-inflammatory cytokines, including interferon-gamma, and tumor necrosis factor-alpha (45-47)(46-47).

## Clinical Variants of MS

Four clinical forms of MS are recognized: primary progressive MS (PPMS), secondary progressive MS (SPMS), relapsing-remitting MS (RRMS) and progressive-relapsing MS (PRMS) (38-48). PPMS patients encompass about 10% of the MS population; their disease is characterized by slow and steady accumulation of neurological deficits from onset without superimposed events. A smaller percentage of patients will have a similar onset but with occasional clinical events (PRMS).

Patients with RRMS have acute exacerbations or clinical events with subsequent variable recovery (remission). At the onset of MS, 80 to 85% of patients will have the RR form of the disease. Fifty percent (50%) of RRMS patients will convert to SPMS within ten years of onset (49), with the peak time of conversion being at about eight years after the onset of the disease (38). The proportion of RRMS progressing to SPMS approaches 80% at twenty-five years. SPMS is characterized by the steady accumulation of significant and persistent neurological deficit with or without superimposed clinical events. Response to IFN-beta therapy may differ based on whether clinical relapses continue to occur in SPMS. The majority of patients with Expanded Disability Status Scores (EDSS) of 6.0 or higher have SPMS.

## Summary of Rebif® Pre-Clinical Trials

Rebif® pre-clinical trials have been reviewed and approved during the registration process and are briefly summarized below.

Rebif® was tested in toxicology studies of up to six months in duration in monkeys and three months in rats and caused no overt signs of toxicity. Rebif® has been shown to be neither mutagenic nor clastogenic. Rebif® has not been investigated for carcinogenicity. A study on embryo/fetal toxicity in monkeys showed no evidence of reproductive disturbances. Based on observations with other alpha and beta interferons, an increased risk of abortions cannot be excluded. No information is available on the effects of Rebif® on male fertility.

## Summary of Rebif® Clinical Trials

Rebif® has been tested in a series of studies in MS subjects at doses ranging from 22 mcg to 132 mcg weekly, with a dosing frequency ranging qw to tiw. The pivotal trial in RRMS was the 6789 (PRISMS) study (34;35;50;51), a double-blind, placebo controlled randomized trial of 22 mcg tiw or 44 mcg tiw Rebif®. The initial two-year study demonstrated that Rebif® at both doses significantly reduced clinical event rate (32% reduction for 44 mcg tiw relative to placebo, p<0.001), slowed disability progression (Hazard Ratio, [HR]=0.63, p=0.01), and reduced MRI lesion activity (77% reduction T2 active lesions, p<0.0001) and accumulation of new lesion burden (-3.8% compared to +11%, p<0.0001). Rebif® is the only disease modifying agent to have demonstrated efficacy on all four of these measures in RRMS.

A test of the importance of treatment regimen was performed in the 21125 (EVIDENCE) study (52), a direct comparative trial of INF-beta-1a 44 mcg tiw (Rebif®) vs. INF-beta-1a 30 mcg qw (Avonex®) in RRMS. This study demonstrated a 12% absolute difference (75% vs. 63%) in the proportion of subjects remaining clinical event-free over twenty-four weeks (primary outcome), which equates to a 19% relative reduction in the risk of clinical event over one year for Rebif® vs. Avonex® subjects (p<0.001). After forty-eight weeks, there was a 10% absolute difference (62% vs. 52%) in the proportion of subjects remaining clinical event-free, which also equates to a 19% relative reduction in the risk of clinical event over one year for Rebif® vs. Avonex® subjects (p=0.009). The time to first clinical event over forty-eight weeks was also significantly reduced with Rebif® (HR=0.70, p=0.003). The main secondary endpoint was based on serial brain MRI showing a 33% reduction in MRI combined unique active lesions for Rebif® relative to Avonex® (p<0.001) over twenty-four weeks, and 36% relative reduction in T2 lesion activity over forty-eight weeks (p<0.001). This study supports the concept of higher, more frequent IFN-beta dosing being more effective than low dose, low-frequency IFN-beta treatment.

## Safety Profile of Rebif® based on Clinical Trials

Rebif® has been well tolerated in all clinical pharmacology studies, even at high doses (up to 66 mcg/m^2^). In later phase clinical trials, Rebif® has been tested across a broad range of doses, for varying duration, and in different stages of MS disease. Dose testing has ranged from 22 mcg to 132 mcg weekly with frequency of administration being daily or thrice weekly. Subject age has ranged from 17 to 68 years.

Additional safety data from clinical trials in all indications studied, post-marketing surveillance and literature reports were compiled in the August 2001 Integrated Summary of Safety that summarized the safety of IFN-beta-1a across several MS and non-MS studies. The majority of exposure is for the specified indication of MS, principally RRMS. The data obtained from this population provide a good understanding of the safety profile of Rebif®.

These data show that, overall, Rebif® is well tolerated at the doses intended for use in the MS population, namely 44 mcg tiw administered by the sc route. The overall mortality rate on treatment is not greater than expected for untreated MS subjects nor is there an excess of serious adverse events on treatment compared to placebo.

The most common adverse events fall into four main categories: injection site reactions (ISRs), flu-like symptoms, hepatic dysfunction and cytopenia. With the exception of ISRs, these events usually occur during the first six months of therapy, are generally mild in severity and usually resolve spontaneously as Rebif® therapy is continued. They rarely require dose reduction or treatment interruption. Other common reactions observed during Rebif® therapy are skin rash, depressive symptoms, and thyroid dysfunction (manifested as hypo- or hyperthyroidism). Details are disclosed in the Rebif® Investigator’s Brochure.

# Trial Objectives

The purpose of this trial is to measure the effects of Rebif® 44 mcg sc tiw on a) remyelination/demyelination, b) lesion and brain volume, c) CNS iron deposition and d) immune status in subjects with RRMS using several MRI techniques.

## Primary Objective

The primary objective is to characterize the effect of Rebif® 44 mcg sc tiw on remyelination/demyelination using a voxel-wise magnetization transfer ratio (VW-MTR) dynamic mapping of normal appearing brain tissue (NABT) in subjects with RRMS over six months of treatment, and with reference to a group of healthy controls (HC).

## Secondary Objectives

Secondary objectives are to characterize the effect of Rebif® 44 mcg on:

- remyelination/demyelination using a VW-MTR dynamic mapping of lesions (T2, T1 & Gd) in RRMS subjects over six months of treatment, and with reference to HC
- brain volume using 3D-T1 imaging of whole brain and of gray matter (GM) in RRMS subjects over six months of treatment, and with reference to HC.
- CNS iron deposition using Susceptibility Weighted Imaging (SWI) of the deep gray matter (DGM), NABT and of the lesions in RRMS subjects over six months of treatment, and with reference to HC.
- Lesion volume (LV) using T2-LV, T1-LV and T1-Gd-LV imaging in RRMS subjects over six months of treatment vs. baseline.

Further secondary objectives are to assess the following immunologic, CNS, and clinical outcomes:

- Immune phenotype of T cells, and monocytes, with special focus on the levels of cytokines involved in the pathogenic Th17 pathway
- Immune cell production of neurotrophic factors, including nerve growth factor (NGF) and brain-derived neurotrophic factor (BDNF), in order to assess potential associations with remyelination, neurodegeneration, iron deposition or GM volumes
- Ferritin and transferrin level assessment, in order to assess the relationship of CNS iron deposition with ferritin levels and with transferrin levels
- Clinical relapses
- Safety evaluation
- Immunologic assessments will help establish associations with CNS effects and clinical outcomes.

# Investigational Plan

## Overall Trial Design and Plan

### Study Design

This is a twenty-four week, two arm, pilot trial to evaluate remyelination, neurodegeneration (brain atrophy), iron deposition in the CNS and immune status with RRMS patients treated with Rebif® 44 mcg sc three times a week (tiw) compared to a HC reference group.

### Primary and Secondary Endpoints

Primary Endpoint

The primary endpoints are the numbers of voxels (counted in milliliters) that change in VW-MTR value (both increase and decrease) from baseline to six-months in NABT of RRMS subjects treated with Rebif® as compared to NABT of HC.

Secondary Endpoints

The secondary MRI endpoints for RRMS subjects treated with Rebif® are:

- the number of voxels that increase in VW-MTR value at three months vs. baseline in NABT
- the increase and decrease in number of voxels from VW-MTR at three- and six-months vs. baseline in lesions
- brain volume using 3D-T1 imaging at three- and six-months vs. baseline in whole brain and GM
- the amount of iron deposition identified via SWI at three- and six-months vs. baseline in DGM, NABT and lesions
- lesion volume from T2-LV, T1-LV and T1-Gd-LV imaging at three- and six-months vs. baseline

The secondary immunology endpoints for RRMS subjects treated with Rebif® (six-months vs. baseline) are:

*Flow cytometry studies on CD4+ and CD8+ gated T-Cells:*

- intracellular IFN-gamma, IL-4, IL-17A, IL-17F, IL-21, IL-22 and IL-10 production
- nerve growth factor (NGF) and brain derived neurotropic factor (BDNF) secretion

*RT-PCR studies on:*

- Magnetic beads separated CD14+ monocytes
  - TLR3, 7 and 9;
  - cytokines IL-1α, IL-1β, IL-23p19, TGF-β, IL-12p70, IL-10, IL-27p28
  - cytokine receptors IL-1R1, IL-12Rβ, IL-23R, IL-21R, and IL-27R.
- Magnetic beads separated CD4+ and CD8+ T-cells
  - transcription factors T-bet, GATA3, ROR, IRF4 and FoxP3;
  - cytokines IFN-gamma, IL-4, IL-17A, IL-17F, IL-21, IL-22 and IL-10;
  - cytokine receptors IL-1R1, IL-23R, IL-21R, IL-12Rβ and IL-27R,
  - neurotrophic factors NGF and BDNF

Other secondary endpoints of the study examine potential associations of the effects of Rebif® in the CNS with peripheral effects on:

- number of clinical relapses
- time to first clinical relapse
- immune cell phenotype: related to CNS infiltration (Th17 cells)
- immune cell production of neurotrophic factors: related to remyelination or preservation of gray matter volume, and
- ferritin and transferrin levels: potentially associated with levels of CNS iron deposition.

### Safety Endpoints

The safety endpoints are to determine the incidence of adverse events following administration of study treatment with Rebif® over six months via electronic case report form (e-CRF) based adverse event collection.

### Study Subjects

It is estimated that fifty five subjects with RRMS will be screened, to achieve twenty-five intent to treat (ITT) subjects. In addition, 15 HC subjects will be enrolled as a reference group according to the same age range requirements and the same ratio of males to females as the RRMS subjects. It is estimated that the 40 subjects (25 RRMS and 15 HC) will be recruited at one center in the USA.

### Study Treatment with Medicinal Product

Subjects who satisfy the entry/eligibility criteria and sign an institutional review board approved Subject Information Sheet and Informed Consent Form will undergo treatment for twenty-four weeks with Rebif®. The dosing regimen complies with the product labeling of the currently marketed formulation of Rebif®.

### Blinding and Randomization

The MRI analysis and immunologic studies will be rater-blinded. Neurologic exams will not be blinded.

### Overall Duration of the Study

Each eligible subject will undergo a screening evaluation, including informed consent, within fourteen days prior to entry into the trial. Study Day 1 is the first day a subject begins his/her injections of Rebif® followed by a twenty-four week course of treatment for RRMS subjects; Study Day 1 is the day of the first MRI measurement for healthy controls. All subjects will have a follow-up telephone call four weeks after study treatment ends in order to monitor their status and well-being.

### Schedule of Assessments

Eligible subjects will undergo a screening assessment within fourteen days prior to entry into the trial. Thereafter, eligible subjects will return to the clinic for assessments on Study Day 1, Week 12, Week 24/Exit and Week 28 (telephone call). Refer to [(See Section 7.2)](#_Assessments_to_be) for a detailed listing of assessments to be performed at each clinic visit and the timing and frequency of those assessments.

The schedule of assessments for the RRMS subjects is illustrated below:

The schedule of assessments for the Healthy Control Group is illustrated below:

## Discussion of Trial Design

This is a twenty-four week, two arm, pilot trial to evaluate remyelination, neurodegeneration (brain atrophy), iron deposition in the CNS and immune status with RRMS patients treated with Rebif® 44 mcg sc tiw compared to a HC reference group.

Subjects who sign an institutional review board (IRB) approved Subject Information Sheet and Informed Consent Form will be enrolled with a scheduled study duration of twenty-four weeks plus a four-week, post-treatment telephone call to evaluate the safety and well-being of the study subjects.

During treatment, RRMS subjects will receive Rebif® 44 mcg sc tiw. MRI measurements for RRMS and HC subjects will be performed at Study Day 1, Week 12 and Week 24/Exit.

## Inclusion of Special Populations

Not applicable.

## Selection of Trial Population

### Inclusion Criteria

The following inclusion criteria must be fulfilled by RRMS subjects:

1. Male and female subjects, 18-65 years of age, inclusive, at the time of informed consent signature
2. RRMS diagnosed according to the McDonald criteria, treatment naïve or currently using any of the FDA-approved DMDs (excluding natalizumab (Tysabri®), mitoxantrone or Rebif®)
3. Have a disease duration for less than ten years
4. Be willing and able to comply with the study procedures for the duration of the trial
5. Have given written informed consent and signed Health Insurance Portability and Accountability Act (HIPAA) Authorization before any study-related activities are carried out
6. Female subjects must not be either pregnant or breast-feeding and must lack childbearing potential, as defined by either:
7. Being post-menopausal or surgically sterile, or using a highly effective method of contraception (defined as a method that results in a low failure rate (i.e. less than 1% per year) when used consistently and correctly, and includes for instance, implants, injectables, combined oral contraceptives, some intrauterine devices, sexual abstinence or vasectomized partner)
8. Female subjects of childbearing potential must have a negative serum pregnancy test at Screening and a negative urine pregnancy test at Study Day 1 to be included in the trial. A urine pregnancy test will also be done at the Week 24/Exit visit

*Note for subjects using a hormonal contraceptive method: No formal drug interaction studies have been carried out with Rebif®. As interferons have been reported to exert an inhibitory activity on hepatic microsomal enzymes, it is unlikely that the clearance of oral contraceptives would increase and result in decreased efficacy. In over 10,000 patient years of clinical trial experience with Rebif®, there has never been any indication of an interaction with oral contraceptives.

The following inclusion criteria must be fulfilled by the Healthy Control subjects:

1. Male and female subjects, 18-65 years of age, inclusive, at the time of informed consent signature
2. Be willing and able to comply with the study procedures for the duration of the trial
3. Have given written informed consent and signed Health Insurance Portability and Accountability Act (HIPAA) Authorization before any study-related activities are carried out
4. Female subjects must not be either pregnant or breast-feeding and must lack childbearing potential, as defined by either:
5. Being post-menopausal or surgically sterile, or using a highly effective method of contraception (defined as a method that results in a low failure rate (i.e. less than 1% per year) when used consistently and correctly, and includes for instance, implants, injectables, combined oral contraceptives, some intrauterine devices, sexual abstinence or vasectomised partner)
6. Female subjects of childbearing potential must have a negative serum pregnancy test at Screening and a negative urine pregnancy test at Study Day 1 to be included in the trial. A urine pregnancy test will also be done at the Week 24/Exit visit

### Exclusion Criteria

Subjects are not eligible for this trial if they fulfill any of the following exclusion criteria:

1. Have received treatment within three months prior to Screening with interferon-beta-1a (Rebif®), IVIG or plasmapheresis
2. Have received treatment within thirty days prior to screening with immunosuppressant agents (e.g. including but not limited to mitoxantrone, cyclophosphamide, cladribine, fludarabine, cyclosporine or total body irradiation) or any other concomitant immunomodulatory therapies (e.g., azathioprine, methotrexate, CellCept®, natalizumab, alemtuzumab/Campathand other immunomodulators/monoclonal agents)
3. Have had a relapse within thirty days prior to the Screening Visit
4. Have received steroid treatment within thirty days prior to the initial MRI scan date at Study Day 1
5. Have inadequate liver function, defined by a alanine aminotransferase (ALT) > 2.5x upper limit of normal (ULN), or alkaline phosphatase > 2.5x ULN, or total bilirubin > 1.5x ULN
6. Have inadequate bone marrow reserve, defined as a total white blood cell count < 3.0x 10^9^/L, platelet count < 75x10^9^/L, hemoglobin < 100g/L
7. Have complete transverse myelitis or simultaneous-onset bilateral optic neuritis
8. Have a history of alcohol or drug abuse
9. Have thyroid dysfunction
10. Have moderate to severe renal impairment
11. Have a major medical or psychiatric illness that in the opinion of the investigator creates undue risk to the subject or could affect compliance with the study protocol
12. Have a history of seizures not adequately controlled by treatment
13. Have serious or acute cardiac disease, such as uncontrolled dysrhythmias, uncontrolled angina pectoris, cardiomyopathy, or uncontrolled congestive heart failure
14. Have, in the opinion of the investigator, any visual, physical or cognitive impairment that would preclude the subject from complying with the study protocol
15. Have a known hypersensitivity or allergy to interferon-beta or any of the excipients
16. Have received an investigational drug or experimental procedure within the past thirty days
17. Are pregnant or attempting to conceive

The following exclusion criteria must be fulfilled by the Healthy Control subjects:

1. Have met any of the above noted criteria

### Exceptions to the Eligibility Criteria

It must be emphasized that the Investigator should make every effort to ensure that all subjects who are offered entry into the trial have satisfied **all** of the inclusion and exclusion criteria outlined above. It is understood, however, that there may be circumstances in which a subject should not necessarily be disqualified for entry into the trial, i.e., if he/she just barely exceeds an exclusionary maximum laboratory result or is slightly less than an allotted timeframe cutoff. In those situations, the Principal Investigator should contact the Sponsor’s Medical Responsible to discuss whether or not to permit enrollment, and such dialogue should be summarized in a written memo for inclusion in the Investigator File and in the Sponsor’s Trial Master File.

### Criteria for Subject Withdrawal

Subjects will be informed that they have the right to withdraw from the trial at any time, without prejudice to their medical care, and are not obligated to state their reason(s). Any withdrawals must be fully documented in the e-CRF and source documents and should be followed-up by the Investigator.

### Withdrawal from the Trial

Subjects are free to discontinue the trial at any time without giving their reasons.

Additionally, the Investigator may withdraw a subject from study treatment at any time if considered to be in the subject’s best interest. Withdrawal from study treatment is mandatory in the following situations:

- Pregnancy [(see Section 7.9.2)](#_Pregnancy_and_In)
- Initiation of treatment with an investigational agent or a prohibited concomitant medication [(see Section 6.4.2)](#_Non-permitted_Medicines)
- Initiation of treatment with another (approved or non-approved) disease modifying therapy for MS
- Common Terminology Criteria for Adverse Events (CTCAE) grade IV lab abnormalities or grade IV ‘related’ serious adverse events [(see Appendix C)](#_Appendix_C-_Common_)
- Adverse events leading to prolonged dose reduction of more than two weeks
- Protocol violations, including non-compliance and lost to follow-up
- Intercurrent illness or significant worsening of previous illness
- Administrative reasons
- Withdrawal of the subject’s consent

In case of premature withdrawal from the study treatment, the assessments scheduled for the Week 24/Exit visit should be performed. [(see Section 7.2.2.3)](#_Week_24/Exit_Visit)

### Replacement of Discontinued Subjects

A subject who fails to meet the protocol-specified criteria for trial entry during the screening period is a screen failure. Screen failures as well as subjects prematurely withdrawn from the trial will not be replaced and subject identification numbers will not be reallocated.

### Follow-up for Discontinued Subjects

If a subject fails to return for follow-up, attempts should be made to contact the subject to determine if the reason for not returning is an adverse event. If a subject withdraws his/her consent to continue in the trial, an attempt must be made to follow-up with the subject.

If treatment is prematurely discontinued, the primary reason for discontinuation must be recorded in the appropriate section of the e-CRF, and all efforts will be made to complete and report the observations as thoroughly as possible. If, for any reason, a subject withdraws or is withdrawn from the trial, every attempt will be made to conduct a follow-up visit with the subject (with all assessments for Week 24/Exit to be completed) [(see Section 7.2.2.3)](#_Week_12/Exit_Visit). Also, all ongoing adverse events will be followed until resolution or at least a period of four weeks ± four days from the last treatment date. After three documented attempts for the four week follow-up telephone call and contact has not been made, subject is considered lost to follow-up.

### Premature Discontinuation of the Trial

The whole trial may be discontinued prematurely in the event of any of the following:

- Sponsor’s decision that continuation of the trial is unjustifiable for medical or ethical reasons
- Slow enrollment of subjects makes it unlikely that the trial will be completed within an acceptable timeframe

If the whole trial is discontinued prematurely, the institutional review board will be informed about the discontinuation of the trial in accordance with applicable regulations.

### Definition of End of Trial

This trial will be considered as completed after:

- all enrolled subjects, who have received at least one dose of Rebif® have completed all scheduled visits (including all prematurely discontinued subjects who should undergo at least the Week 24/Exit visit and the four-week, post-treatment telephone call);
- follow-up of all subjects with ongoing adverse events at the conclusion of treatment has been addressed; [(see Section 7.9.1.6)](#_Monitoring_of_Subjects)
- all data discrepancies have been resolved;
- the database has been locked; and
- the study site has undergone a final closure visit (usually four to six weeks after the database lock).

### Medical Care of Subjects after End of Trial

After a subject has completed the trial or has withdrawn early, usual treatment will be administered in accordance with the study site’s standard of care and generally accepted medical practice and depending on the subject’s individual medical needs. Rebif® will not be provided to subjects after the conclusion of the trial. No extension trial is planned.

# Medicinal Product(s) and Other Drugs Used in the Trial

The term “Medicinal Product” refers to Rebif® 44 mcg tiw for sc injection.

## Dosage and Administration

**Rebif®**

Rebif® will be supplied in pre-constituted syringes. During the four week titration phase of Rebif®, dosages must be adjusted according to the following dose titration schedule (refer to the locally approved package insert for the administration, storage and handling instructions for Rebif®):

| **Week** | **Recommended Titration** | **Volume** | **IFN-beta-1a dose** |
| --- | --- | --- | --- |
| 1-2 | 20% | 0.1 mL | 8.8 mcg |
| 3-4 | 50% | 0.25 mL | 22 mcg |
| 5 + | 100% | 0.5 mL | 44 mcg |

Source: Rebif® Package Insert: Schedule for Rebif® dose titration.

## Packaging and Labeling

Packaging and labeling will be in accordance with applicable local regulatory requirements and applicable Good Manufacturing Process Guidelines.

## Preparation, Handling and Storage

On site, all Rebif**®** must be stored in a secure location, preferably in a temperature controlled locked refrigerator or cold room, and may be dispensed only by the Investigator or by a member of staff specifically authorized by the Investigator, or by a pharmacist, as appropriate. Any deviations from the recommended storage conditions should be immediately reported to the Sponsor and use of the Medicinal Product interrupted until authorization for its continued use has been given by the Sponsor.

Rebif® should be stored between 2°C and 8°C (36°F and 46°F) and protected from light. DO NOT FREEZE.

Please refer to the locally approved package insert for storage and handling instructions of Rebif®.

## Concomitant Medications and Therapies

### Permitted Medicines

Any concomitant medications (other than those excluded by the protocol) that are considered necessary for the subjects’ welfare and will not interfere with the Medicinal Product may be given at the Investigator’s discretion (e.g. analgesics for treatment of flu-like symptoms and corticosteroids for the treatment of relapses).

All such therapy will be recorded in the e-CRF from the time the Informed Consent Form is signed until the final post-treatment follow-up, along with medication name, dosage information, dates of administration, and reasons for use.

Concomitant medications for the following reasons should be documented in the e-CRF:

- MS related condition (e.g. pain, fatigue, bladder dysfunction, spasticity, etc.)
- Medical conditions reported in the subject’s Medical History (e.g. pain, headaches, depression, etc.)
- Prophylactic Use (e.g. flu-like symptoms, etc.)
- Self-medication (e.g. vitamins, nutritional supplements, herbal/natural products, etc.)

### Non-permitted Medicines

In line with the exclusion criteria in [(see Section 5.4.2)](#_Exclusion_Criteria), subjects may not use any investigational medications or any of the following therapies during the trial:

- Cytokine or anti-cytokine therapy
- Any interferons other than Rebif®
- Intravenous immunoglobulin (IVIg)
- Monoclonal antibodies, mitoxantrone, cytotoxic or immunosuppressive therapy including but not limited to cyclophosphamide, cyclosporin, methotrexate, azathioprine, linomide, teriflunomide, laquinimod, cladribine, total lymphoid irradiation, anti-lymphocyte monoclonal antibody treatment (e.g., natalizumab, alemtuzumab/Campath, anti-CD4)
- Chronic or monthly pulse corticosteroids
- Plasmapheresis
- Glatiramer acetate
- Telbivudine

If the administration of a non-permitted concomitant medication becomes necessary during the trial, the subject should be withdrawn from the trial. [(see Section 5.4.4)](#_Criteria_for_Subject)

Any additional concomitant therapy that becomes necessary during the trial and any change to concomitant drugs must be recorded in the corresponding section of the e-CRF, noting the name, dose, duration and indication of each drug.

### Concomitant Procedures

Additionally, any non-drug therapies or diagnostic, therapeutic, or surgical procedures performed during the trial will be recorded in the e-CRF, including the date and reason for procedure.

### Special Precautions

Not Applicable

## Medicinal Product Accountability

The Investigator is responsible for ensuring drug accountability, including reconciliation of drugs and maintenance of drug records.

- Upon receipt, the Investigator (or designee) will check for accurate delivery and acknowledge receipt by signing (or initialing) and dating the documentation provided by the Sponsor and returning it to the Sponsor. A copy will be retained for the Investigator File.
- The dispensing of drug will be carefully recorded on appropriate drug accountability forms and an accurate accounting will be available for verification by the Sponsor Monitor at each monitoring visit.
- Drug accountability records will include:
- Confirmation of drug delivery to the trial site.
- The inventory at the site of drug provided by the Sponsor and prepared at the site.
- The use of each dose by each subject.
- The return to the Sponsor or alternative disposition of unused drug.
- Dates, quantities, batch numbers, expiry dates and (for drug prepared at the site) formulation, as well as the subjects’ trial numbers.
- The Investigator should maintain records that adequately document:
- That the subjects were provided the doses specified by the clinical trial protocol/amendment(s), and
- That all drug provided by the Sponsor was fully reconciled.

Unused drug must not be discarded or used for any purpose other than the present trial. Medicinal Product that has been dispensed to a subject must not be re-dispensed to a different subject.

The Sponsor Monitor will periodically collect the drug accountability form and will check all returns (both unused **and** used containers) before arranging for their return to the Sponsor or authorizing their destruction by the trial site.

## Assessment of Medicinal Product Compliance

Subjects should be routinely encouraged by the study personnel to administer all doses of medications over the entire twenty-four weeks of treatment.

Subject compliance will be evaluated by the following:

- Documentation of missed injections in the Study Treatment Administration e-CRF
- Review of diary cards, on which the subjects will enter the dates and times of each of their injections
- Subjects should be instructed to bring with them to each visit both opened and unopened drug packages, in order to allow the assessment of compliance with trial treatment. Drug administration must be recorded in the e-CRF, as applicable.

## Method of Blinding

This trial is open-label. The MRI analysis and immunologic studies will be rater-blinded. Neurologic exams will not be blinded.

## Emergency Unblinding

Not applicable.

## Treatment of Overdose

An overdose is defined as any dose greater than the highest daily dose included in the clinical trial protocol. Any overdose must be recorded in the trial medication section of the e-CRF.

For monitoring purposes, any case of overdose – whether or not associated with an adverse event (serious or non-serious) – must be reported to the Sponsor’s Global Drug Safety department in an expedited manner using the appropriate reporting form [(see Section 7.9.1.11)](#_Procedure_for_Reporting).

## Medical Care of Subjects after End of Trial

After a subject has completed the trial or has withdrawn early, usual treatment will be administered, if required, in accordance with the trial site’s standard of care and generally accepted medical practice and depending on the subject’s individual medical needs.

# Trial Procedures and Assessments

## Schedule of Assessments

Prior to performing any trial assessments not part of the subject’s routine medical care, the Investigator will ensure that the subject or the subjects legal representative has provided written informed consent according to the procedure described in Section 9.2.

A screening log will be completed for all subjects who sign the Informed Consent Form (ICF) but do not subsequently enter the trial. Subjects will be identified by their initials and dates of birth, and reason(s) for exclusion from the trial will be recorded.

Please refer to [(see Appendix A)](#_Appendix_A_-) for a Schedule of Assessments to be performed during the conduct of this trial, as well as the frequency of testing over twenty-four weeks of treatment and the four-week, post-treatment telephone call.

In summary, subjects who sign an institutional review board approved ICF and who satisfy the eligibility criteria at Screening should then return to the clinic on Study Day 1, Week 12 and Week 24/Exit. The subjects will also receive a telephone call four weeks post-treatment to monitor their status and well-being.

Due to the workload of the clinic, as well as subjects’ own personal schedules, it is understood that there may be conflicts in subjects returning to the clinic on the exact day of their specified contacts. As such, a “window” of ± seven days is permitted for subjects to be evaluated at each scheduled contact (Study Day 1, Week 12, Week 24 and Week 28 (telephone call).

Subjects will be asked to keep diary cards to collect information regarding treatment administration, concomitant medications, concomitant procedures and any adverse event(s) they experienced. This information will be discussed with each subject, and once confirmed by the Investigator or study personnel, it will be recorded in the appropriate e-CRF.

## Assessments to be performed at each scheduled visit

The assessments to be performed and/or information to be obtained at each respective clinic visit or telephone contact are as follows:

### Screening Period

**RRMS Subjects**

The following information should be obtained at Screening (within 14 days prior to Study Day 1):

- A signed institutional review board (IRB) approved ICF and HIPAA Authorization (provide a copy to the subject, as well as retain a copy for the Investigator File).
- Medical History, including:
- Demographic data, including date of birth, gender and race
- Review of eligibility criteria
- Relevant medical history, including past and ongoing medical conditions related to MS
- For female subjects, birth control methods will be discussed and recorded
- Recording of adverse events and concomitant medications/procedures
- Physical examination unrelated to MS, including height, weight and vital signs [(see Section 7.2.6.1)](#_Physical_Examinations)
- Neurological assessment (full exam including EDSS) [(see Section 7.2.6.2)](#_Neurological_Assessments)
- Laboratory Assessments (Chemistry, Hematology, ferritin, transferrin, uric acid)
- A blood sample will be collected for analysis of thyroid function tests.
- Serum pregnancy test for female subjects of childbearing potential

**NOTE: Confirmation that a subject is not pregnant must be confirmed by a negative serum beta human chorionic gonadotropin (hCG) test within 14 days prior to Study Day 1 (the first day of dosing) and by a negative urine pregnancy test on Study Day 1.**

**Healthy Control Subjects**

The following information should be obtained at Screening (within 14 days prior to Study Day 1):

- A signed institutional review board (IRB) approved ICF and HIPAA Authorization (provide a copy to the subject, as well as retain a copy for the Investigator File).
- Medical History, including:
- Demographic data, including date of birth, gender and race
- Review of eligibility criteria
- Relevant medical history, including past and ongoing medical conditions
- Recording of adverse events and concomitant medications/procedures
- Physical examination unrelated to MS, including height, weight and vital signs [(see Section 7.2.6.1)](#_Physical_Examinations)
- Neurological assessment (full exam, not including EDSS) [(see Section 7.2.6.2)](#_Neurological_Assessments)

### Treatment Period

The treatment period begins with the completion of all baseline evaluations and the initiation of study treatment on Study Day 1 and continues through to the completion of the treatment period at the Week 24/Exit visit.

#### Study Day 1

**RRMS Subjects**

The following assessments must be performed:

- Re-confirmation that eligibility criteria are satisfied
- For female subjects of childbearing potential, a urine pregnancy test, to be performed locally at the study site
- Blood collection for Immunological Samples
- MRI
- Injection Training/Dispensing of Rebif®
- Provide each subject with a diary card and train on proper completion to record injection dates/times, concomitant medications/procedures and adverse events

**Healthy Control Subjects**

The following assessments must be performed:

- Provide each subject with a diary card and train on proper completion to record adverse events and concomitant medications/procedures
- Blood collection for Immunological Samples
- For female subjects of childbearing potential, a urine pregnancy test, to be performed locally at the study site
- MRI

#### Week 12 Visit

**RRMS Subjects**

The following procedures and assessments will be performed at the Week 12 visit:

- Recording of adverse events and concomitant medications/procedures
- Laboratory Assessments (Chemistry, Hematology, ferritin, transferrin, CBC, LFTs, platelets, uric acid)
- Physical examination unrelated to MS, including height, weight and vital signs [(see Section 7.2.6.1)](#_Physical_Examinations)
- Neurological assessment (full exam, not including EDSS) [(see Section 7.2.6.2)](#_Neurological_Assessments)
- MRI
- Dispensing of Rebif®
- Dispensing of subject take-home diary (for recording injections, adverse events, concomitant medications/procedures for next six weeks)

**Healthy Control Subjects**

The following assessments must be performed:

- Recording of adverse events and concomitant medications/procedures
- Physical examination unrelated to MS, including height, weight and vital signs [(see Section 7.2.6.1)](#_Physical_Examinations)
- Neurological assessment (full exam, not including EDSS) [(see Section 7.2.6.2)](#_Neurological_Assessments)
- Dispensing of subject take-home diary (for recording adverse events, concomitant medications/procedures for next six weeks
- MRI

#### Week 24/Exit Visit

**RRMS Subjects**

The following procedures and assessments will be performed at the Week 24/Exit visit or following early termination of a subject from the trial:

- Recording of adverse events and concomitant medications/procedures
- Laboratory Assessments (Chemistry, Hematology, ferritin, transferrin, CBC, LFTs, platelets, uric acid)
- A blood sample will be collected for analysis of thyroid function tests.
- Physical examination unrelated to MS, including height, weight and vital signs [(see Section 7.2.6.1)](#_Physical_Examinations)
- Neurological assessment (full exam, not including EDSS) [(see Section 7.2.6.2)](#_Neurological_Assessments)
- Blood collection for Immunological Samples
- MRI
- For female subjects of childbearing potential, a urine pregnancy test, to be performed locally at the study site
- Reason for termination (if subject terminates early)
- Drug accountability

**Healthy Control Subjects**

The following assessments must be performed:

- Recording of adverse events and concomitant medications/procedures
- Physical examination unrelated to MS, including height, weight and vital signs [(see Section 7.2.6.1)](#_Physical_Examinations)
- Neurological assessment (full exam, not including EDSS) [(see Section 7.2.6.2)](#_Neurological_Assessments)
- For female subjects of childbearing potential, a urine pregnancy test, to be performed locally at the study site
- MRI

### Post-Treatment Period

#### Week 28 (four-week, post-treatment telephone call)

The following assessments will be made during a follow-up telephone call or clinic visit for both RRMS subjects and the Healthy Control Group four weeks after the Week 24/Exit visit:

- Recording of adverse events and concomitant medications/procedures

Subjects who withdraw prematurely before the Week 24/Exit visit, especially if due to an adverse event(s), should be contacted by telephone four weeks after the early withdrawal date to undergo the required follow-up assessments. If after three documented attempts for the four week follow-up telephone call and contact has not been made, subject is considered lost to follow-up.

### Laboratory Assessments

The subject may be either fasting or non-fasting for the following laboratory assessment to be performed at Screening, Week 12 and Week 24/Exit visit:

- Standard clinical laboratory assessments, including blood chemistries, hematology (CBC with Differential), and urinalysis
- Special chemistries: ferritin, transferrin, uric acid
- Serum pregnancy test for female subjects of childbearing potential (Screening only)
- Urine pregnancy test for female subjects of childbearing potential (Study Day 1 and Week 12/Exit)

All tests will be performed by a local laboratory. [See Appendix B - Laboratory Parameters](#_Appendix_B_-_) for the specific analytes to be measured).

### Immunological Assessments

Immunological samples will be taken on Study Day 1 for RRMS and HC subjects. Immunological samples will be taken on Week 24/Exit Visit for RRMS subjects only (for specific information on samples [see section 5.1.2](#_Primary_and_Secondary)):

The preparation and handling of blood samples for immunological testing, including the collection, labeling, storage and shipment requirements will be supplied in a separate instruction manual.

### Other Assessments

Not applicable.

#### Physical Examinations

A physical examination unrelated to MS, for RRMS subjects, will be done at Screening and at the Week 24//Exit visit and will include height, weight and vital signs (blood pressure, pulse, respiration rate and temperature). A general physical exam for the HC subjects will be done at Screening and at the Week 24/Exit visit to rule out any abnormalities. The results will be recorded in the e-CRF as normal or abnormal. If the initial examination is abnormal, and the abnormality occurred prior to informed consent, it should be captured in the medical history form of the e-CRF. If the abnormality occurred or worsened after informed consent, including abnormalities detected at the Week 24/Exit visit, it should be captured in the adverse event form of the e-CRF.

#### Neurological Assessments

A complete neurological assessment for RRMS subjects will be done at Screening and at the Week 24/Exit visit. The full exam will include mental status, cranial nerve status, motor exam, sensory exam, coordination and gait. The EDSS will only be scored for RRMS subjects at the screening visit.

If the initial examination is abnormal, and the abnormality occurred prior to informed consent, it should be captured in the medical history form of the e-CRF. If the neurological signs, including relapses, occurred or worsened after informed consent, including signs detected at the Week 24/Exit visit, they should be captured in the adverse event form of the e-CRF.

#### MRI Assessments

Neuroimaging assessments for both RRMS and HC subjects will be done at Study Day 1, Week 12 and the Week 24/Exit visit.

##### MRI Acquisition

MRI exams of the brain will be performed on a 3T GE Signa LX Excite 12.0 scanner at the Buffalo Niagara MRI Center, located within Buffalo General Hospital, 100 High St., Buffalo, NY. In addition to localizer/scout images and calibration images, the following scans will be acquired: dual echo proton density and T2-weighted image (WI), 3D-inversion recovery fast-spoiled-gradient recalled (IR-FSPGR), T1-WI, spin-echo (SE) T1-WI with and without gadolinium (Gd) contrast, fast attenuated inversion recovery (FLAIR), gradient echo (GRE) T1-weighted magnetization transfer imaging (MTI) with and without saturation pulse and susceptibility weighted imaging (SWI). Conventional 2D scans (PD/T2, FLAIR, and T1 SE pre- and post-contrast) will be acquired with consistent voxel size (64 slices 3mm slice thickness, with FOV = 25.6, matrix = 256 x 256 and phase FOV =.75), and slice selection (oblique axial images parallel to the subcallosal plane). MTI will be acquired with 4mm slice thickness, FOV = 25.6, phase FOV = .75, matrix 256x256, TR = 50, TE = 6 and FA = 10. 3D GRE SWI will be acquired with 2mm slice thickness, FOV 25.6, matrix 512x256, phase FOV = .75.

Patients with MS will receive a single dose (0.1 mmol/kg) gadolinium contrast injection prior to the FLAIR scan, which will be immediately followed by the T1 SE post contrast scan. The FLAIR scan is unaffected by the contrast and is used to provide a consistent time delay between contrast injection and post-contrast imaging. Normal control participants will not receive contrast injection.

All participants will be positioned in the scanner to maximize comfort and minimize head movement. The nasion landmark will be used to position the head in the isocenter of the magnetic field.

##### MRI Analysis

Image analysis will be performed at the Buffalo Neuroimaging Analysis Center (BNAC). The operators will be blinded to patients’ clinical characteristics and clinical status. Analyses will be performed on a Red Hat Enterprise Linux workstation (Red Hat, Inc. Raleigh, NC, USA). At all stages of manual, semi-automated and automated analyses, quality-control montage imaging output analyses files will be examined by an expert observer.

*Quantitative Lesion Analysis*: Although lesion activity is not a primary outcome of the study, T2-, T1- and Gd lesion analyses will be completed on all scans to generate lesion masks that will be required by the SWI and VW-MTR analyses. Calculation of the lesion volumes (Gd, T2 and T1) is based on semi-automated tracing on computer displayed images by trained research clinical trial operators. The analyses are confirmed (accepted) by medical doctors and/or certified clinical neuroimagers. Analysis discrepancies found between neuroimagers and operators are resolved via review by the BNAC Director. The data are analyzed using JIM (version 4.0, Xinapse Systems, Northants, UK, http://www.xinapse.com).

*T2-LV:* A T2 lesion is defined as a rounded or oval hyperintense area in normal appearing brain tissue (NABT), according to the guidelines by Filippi (14).

For analysis of hyperintense LV on T2-weighted images, use of FLAIR scans (which show higher sensitivity and lower inter-observer variability than fast spin-echo T2-weighted images in the detection of areas of T2 prolongation in MS) will be used. This method of quantifying the total brain hyperintense and hypointense LVs has been previously described in detail (53). Briefly, the T2-LV is calculated using a highly reproducible semiautomated local thresholding technique for lesion segmentation. The T2 lesions are outlined on FLAIR images on each axial slice (proton density and T2-weighted scans are always used to increase confidence in lesion detection). The mean COV for T2-LV was 1.1% (range 0.9 to 3.5) for inter-observer reproducibility and 1.5% (range 1.0 to 4.1) for intra-observer reproducibility.

*T1-LV:* A conservative approach is used for calculation of lesions on T1-WI. A hypointense lesion is defined as any region visible on the CSE T1-weighted sequence with a low signal (short TR, short TE) intensity between those of the CSF and GM, and corresponding to a region of high signal intensity on the T2-weighted sequence (long TR, long TE).

Lesions are delineated as ROIs and the volume will be calculated simply for each sequence by multiplying the total ROI area by the slice thickness. Results are expressed in milliliters (ml). The mean COV for T1-LV was 2.5% (range 1.4 to 5.5) for inter-observer reproducibility and 3.2% (range 1.8 to 5.5) for intra-observer reproducibility.

*Gd-LV:* A Gd-enhancing lesion is defined as a typical area of hyperintense signal on post contrast T1-WI according to the guidelines by Barkhof (54) that is not hyperintense on a T1-weighted precontrast image.

The volume of Gd-enhancing lesions on post-contrast T1-WI is determined in a similar manner as T2-LV above, and has been previously described (55)

*Brain volume:* Gray matter atrophy will be measured by comparing baseline and six-months MRI scans using SIENAX (Structural Image Evaluation, using Normalization, of Atrophy, cross-sectional; (56) part of FSL (57). With SIENAX brain volumes will be normalized and segmented into three classes: GM, WM, third ventricular width and CSF. Lesion masks will be used to remove any lesion-related voxels misclassified as gray matter.

*Voxel-wise MTR*: The VW-MTR analysis will be completed in nine steps: 1) Lesions will be identified on T2-FLAIR, T1 pre-contrast (black holes) and T1 post-contrast (gadolinium enhancing) images (as described above). 2) MTR and accompanying T1, T1+Gd, FLAIR and SPGR images will be coregistered using FLIRT (FMRIB, Oxford). 3) 3DT1-weighted images (IR-FSPGR) will be segmented to classify voxels as gray matter, white matter and cerebral spinal fluid. Lesions will be corrected using a combination of tools available from FMRIB. 4) MTR maps will be generated by a voxel-wise application of the standard MTR formula (((M_0_ – M_S_)/M_0_) x 100). 5) Voxel-wise MTR difference maps will be created by subtracting MTR map pairs based on longitudinal time points. 6) TFCE will be applied to the difference maps to increase classification sensitivity. 7) A Monte Carlo process will be used to derive meaningful statistical values to be associated with the TFCE generated clusters. 8) Voxels will be classified as changing or not changing and the volume of changing tissue will be calculated by multiplying the number of changing voxels by the individual voxel volume. Lesion maps will be overlaid to quantify increasing and decreasing MTR in whole-brain, NABT, T2, T1 and T1+Gd lesion volumes. 9) Image quality will be checked at every stage new images are generated, beginning with the quality control of the raw input images.

*SWI:* First, SWI phase and magnitude images will be reconstructed from raw data using a channel re-centering and normalization process in order to ensure proper composition of multi-channel data. The phase image will then be high-pass filtered using a 64x48 Gaussian window in the center of k-space in order to yield a strongly iron-sensitive phase map. Correction for geometric field-induced distortions will be carried out via a non-linear image unwarping technique. These images will then be co-registered into a corresponding subject-specific high-resolution upsampled FLAIR space using a rigid-body linear image registration technique addition, the phase image will be linearly transformed and thresholded to create a phase mask. This phase mask will then be composited with the magnitude image to create an SWI weighted image as described in (30).

Veins will be identified on the phase map via a multi-scale line filtering approach. Voxels will be classified as vein if they exhibit significant local linearity within a 3-dimensional neighborhood. Some additional filtering (Canny edge and intensity-based) will also be applied in a weighted manner to provide for the cleanest possible outcome image. This will result in a “soft” classification map, which will then be binarized to provide a whole-brain vein mask.

T2 lesions and T1 black holes will be identified and delineated using a previously described semi-automated edge contouring technique (53). A similar technique will be used to identify SWI magnitude-visible lesions and SWI phase-visible lesions. Phase-visible lesions will be further subdivided into nodular, ring-shaped, and scattered categories, and will also be marked as probable or definite. All lesion masks will be co-registered into the subject-specific upsampled FLAIR space, at which point spatial overlap maps will be calculated. These will be used to calculate mean magnitude and phase values for each lesion type and each intersection of lesion types. In addition, the whole-brain vein mask will be used to ensure that mean values are calculated only over non-vein voxels.

The high-resolution T1-weighted image will then be segmented and parcellated into various tissue classes and regions. Subcortical gray matter structures will be segmented using a combination of semi-automated edge-contouring and fully-automated model-based registration approaches specifically, thalamus, caudate, putamen, globus pallidus, hippocampus, amygdala, and nucleus accumbens will be identified automatically, and the red nucleus, pulvinar nucleus of the thalamus, and substantia nigra will be identified semi-automatically. All regions will be identified separately in each hemisphere. In addition, the image will be deskulled (56) and segmented into tissue-specific compartments via an expectation maximization optimized hidden Markov random field statistical model (58). As part of this segmentation process, T2 and T1 lesion masks will also be co-registered into the space of the high-resolution T1 and used to correct any lesion misclassification due to T1 hypointensity. The lesion masks will also be used to distinguish between whole brain (gray matter and white matter) tissue compartments and normal-appearing tissue compartments. As with the lesion measurements, mean non-vein phase and magnitude values will be calculated for each subcortical region and each tissue compartment.

## Pharmacokinetics

Not applicable.

## Body Fluid(s)

During the conduct of this trial, blood and urine samples will be obtained to measure chemistry, hematology, urinalysis and special chemistry tests including ferritin and transferrin. Blood and urine pregnancy testing will be done for females of childbearing potential at Screening, Study Day 1 and at the Week 24/Exit visit. Please refer to [Section 7.2](#_assessments_to_be) for a schedule of when the laboratory assessments will be performed and [Appendix B - Laboratory Parameters](#_Appendix_B_-_) for a listing of laboratory parameters.

## Pharmacokinetic Calculations

Not applicable.

## Biomarkers/Pharmacogenetics (PGx)

Not applicable.

## Other Assessments

Not applicable.

## Assessment of Efficacy

Efficacy will be assessed through MRI measures (see section [(see section 5.1.2)](#_Primary_Endpoint)

## Assessment of Safety

The following safety parameters will be evaluated as part of the safety assessment (from signing the informed consent through the four-week, post-treatment period):

- Time on treatment and study
- Premature termination and reasons for premature termination from treatment
- The incidence, severity, and relationship to study medication of treatment emergent adverse events
- Serious adverse events
- Clinically significant changes in laboratory tests and worst toxicity according to the Common Terminology Criteria for Adverse Events (CTCAE) toxicity grading criteria [(see Appendix C)](#_Appendix_C-_Common_)
- Concomitant medication use
- Physical examination
- Vital signs (including blood pressure and heart rate)

A comprehensive assessment of any apparent toxicity experienced by a subject will be performed throughout the course of the trial, from the time of the subject’s signature of informed consent. Study personnel will report any adverse event, whether observed by the Investigator or reported by the subject. The reporting period for adverse events is described in [(See Section 7.9.1.5)](#_Definition_of_the).

### Adverse Events

#### Adverse Event Definitions

An adverse event (AE) is any untoward medical occurrence in a subject or clinical investigation subject administered a pharmaceutical product, which does not necessarily have a causal relationship with this treatment. An AE can therefore be any unfavorable and unintended sign (including an abnormal laboratory finding), symptom, or disease temporally associated with the use of Rebif®, whether or not considered related to Rebif®.

In cases of surgical or diagnostic procedures, the condition/illness leading to such a procedure is considered as the AE rather than the procedure itself.

In case of a fatality, the cause of death is considered as the AE, and the death is considered as its outcome.

#### Eliciting Adverse Events

Data on AEs will be obtained at scheduled and unscheduled clinic visits, based on information spontaneously provided by the subject and/or through questioning of the subject. AE data may also be obtained from subject diary cards, but such information must be reviewed with the subject and assessed medically before it is transcribed onto the e-CRF.

To elicit AEs, questioning at each clinic visit should begin with simple non-leading

questions. For example:

- How have you felt since your last visit?
- Have you had any health problems since you were here last?

If a subject is seen by a physician not involved with the trial in relation to an AE, the Investigator should make every effort to contact the treating physician in a timely manner in order to obtain all information necessary for appropriate reporting of the event.

#### Grading the Severity of an Adverse Event

The Investigator is required to grade the severity/intensity of each AE according to the Qualitative Toxicity Scale, as follows:

**Mild**: The subject is aware of the event or symptom, but the event or symptom is easily tolerated.

**Moderate**: The subject experiences sufficient discomfort to interfere with or reduce his or her usual level of activity.

**Severe**: Significant impairment of functioning: the subject is unable to carry out usual activities.

#### Grading the Relationship of an Adverse Event

Investigators must also systematically assess the causal relationship of AEs to the Medicinal Product using the following definitions, with the decisive factor being the temporal relationship between the AE and administration of the Medicinal Product:

**Probable:** A causal relationship is clinically/biologically highly plausible and there is a plausible time sequence between onset of the AE and administration of the drug and there is a reasonable response on withdrawal.

**Possible:** A causal relationship is clinically/biologically plausible and there is a plausible time sequence between onset of the AE and administration of the drug.

**Unlikely:** A causal relationship is improbable and another documented cause of the AE is most plausible.

**Unrelated:** A causal relationship can be definitively excluded and another documented cause of the AE is most plausible.

#### Definition of the Adverse Event Reporting Period

The AE reporting period for safety surveillance begins when the subject is included into the trial (date of first signature of informed consent) and continues through the subject’s four-week, post-treatment telephone call.

#### Monitoring of Subjects with Adverse Events

Any AE that occurs during the course of a clinical trial and is considered to be possibly related to the Medicinal Product must be monitored and followed up until the outcome is known, unless the subject is documented as “lost to follow-up”. Reasonable attempts to obtain this information must be made and documented. It is the responsibility of the Investigator to ensure that any necessary additional therapeutic measures and follow-up procedures are performed.

#### Abnormal Laboratory Findings and Other Abnormal Investigational Findings

Abnormal laboratory findings and other abnormal investigational findings should not be reported as AEs unless they are associated with clinical signs and symptoms, lead to treatment discontinuation or are considered otherwise medically important by the Investigator. If an abnormality fulfills these criteria, the identified medical condition (e.g. anemia, increased ALT) must be reported as the AE rather than the abnormal value itself.

#### Definition of Serious Adverse Event

A serious adverse event (SAE) is any untoward medical occurrence that at any dose:

- Results in death.
- Is life-threatening.

NOTE: The term “life-threatening” in this definition refers to an event in which the subject is at risk of death at the time of the event; it does not refer to an event that hypothetically might cause death if it were more severe.

- Requires inpatient hospitalization or prolongation of existing hospitalization.
- Results in persistent or significant disability/incapacity.
- Is a congenital anomaly/birth defect.
- Is otherwise considered as medically important.

Important medical events that may not result in death, be life-threatening, or require hospitalization may be considered as SAEs when, based upon appropriate medical judgment, they may jeopardize the subject or may require medical or surgical intervention to prevent one of the outcomes listed in this definition. Examples of such events include allergic bronchospasm requiring intensive treatment in an emergency room or at home, blood dyscrasias or convulsions that do not result in inpatient hospitalization, or the development of drug dependency or drug abuse.

For the purposes of reporting, any suspected transmission of an infectious agent via a Medicinal Product is also considered a SAE and all such cases should be reported in an expedited manner as described in [(see Section 7.9.1.11)](#_Procedure_for_Reporting).

#### Events that Do Not Meet the Definition of an SAE

Elective hospitalizations to simplify trial treatment or trial procedures (e.g. an overnight stay to facilitate chemotherapy and related hydration therapy application) are not considered as SAEs. However, all events leading to unplanned hospitalizations or unplanned prolongation of an elective hospitalization (e.g., undesirable effects of any administered treatment) must be documented and reported as SAEs.

#### Events Not to Be Considered as AEs/SAEs

Medical conditions present at Screening that do not worsen in severity or frequency during the trial are defined as baseline medical conditions, and are NOT to be considered AEs. These conditions should be adequately documented on the medical history form of the e-CRF. However, baseline conditions that worsen in severity or frequency during the trial should be reported as AEs.

#### Procedure for Reporting Serious Adverse Events

In the event of any new SAE occurring during the reporting period, the Investigator must immediately **(i.e. within a maximum 24 HOURS after becoming aware of the event)** inform the Sponsor by telephone, by fax or by e-mail.

When an event (or follow-up information) is reported by telephone, a written report must be sent immediately thereafter to the Sponsor by fax or e-mail.

Reporting procedures and timelines for follow-up information are the same for any new information on a previously reported SAE.

Once notified of a possible SAE, the Investigator/Reporter must complete the SAE Report, following specific instructions (i.e., the SAE Report Completion Instructions to be provided by the Sponsor) and send it directly to the Sponsor’s Global Drug Safety department by electronic mail or fax, using the dedicated e-mail address and fax numbers specified below:

e-mail: [GlobalDrugSafety@merckserono.net](mailto:GlobalDrugSafety@merckserono.net)

fax: +800 72 33 8932 (international toll-free number)

+1 781 681 2392 (US sites)

For any new SAEs, the following minimum information is required in the initial notification of the Sponsor:

- Clear identification of the Investigator/Reporter, with full contact information;
- Subject identification details (protocol number, site number, subject’s initials, and date of birth);
- Medicinal Product administration details (doses and dates);
- Diagnosis of the event, with the description (or a brief description of signs/symptoms/clinical course, if the diagnosis is not available) and the date of onset;
- Reason(s) for considering the event serious; and
- Relationship of the event with the drug or with the trial procedure (i.e., the causality according to the Investigator) [(see Section 7.9.1.4)](#_Grading_the_Relationship).

All written reports should be transmitted on the SAE Report, which must be completed by the Investigator following specific completion instructions. Relevant pages from the e-CRF may be provided in parallel (e.g., medical history, concomitant medications). In all cases, the information provided in the SAE Report must be consistent with the data on the event that are recorded in the corresponding sections of the e-CRF.

The Investigator/Reporter must respond to any request for follow-up information (e.g. additional information, outcome and final evaluation, specific records where needed) or to any question the Sponsor may have on the SAE within the same timelines as described for initial reports. This is necessary to permit a prompt assessment of the event by the Sponsor and, as applicable, to allow the Sponsor to meet strict regulatory timelines associated with expedited safety reporting obligations.

Requests for follow-up will usually be made by the responsible Monitor, although in exceptional circumstances the Global Drug Safety department may contact the Investigator directly to obtain clarification or to discuss a particularly critical event.

#### Safety Reporting to Regulatory Authorities, Investigators and Institutional Review Boards

The Sponsor will send appropriate safety notifications to regulatory authorities in accordance with applicable laws and regulations.

The Investigator must comply with any applicable institution-specific requirements related to the reporting of SAEs (and in particular deaths) involving his/her subjects to the institutional review board (IRB) that approved the trial.

In accordance with ICH/GCP guidelines, the Sponsor will inform the Investigator of “findings that could adversely affect the safety of subjects, impact the conduct of the trial or alter the IRB’s approval/favorable opinion to continue the trial.” In particular and in line with respective regulations, the Sponsor will inform the Investigator of AEs that are both serious and unexpected and are considered to be related to the administered product (“suspected unexpected serious adverse reactions” or SUSARs). The Investigator should place copies of these safety reports in the Investigator File. National regulations with regard to safety report notifications to Investigators will be taken into account.

The Investigator will be responsible for promptly notifying the concerned local IRB of any safety reports provided by the Sponsor and of filing copies of all related correspondence in the Investigator File.

#### Methods of Recording Adverse Events in the e-CRF

Complete, accurate and consistent data on all AEs experienced for the duration of the reporting period will be reported on an ongoing basis in the appropriate section of the e-CRF. Among these AEs, all SAEs must be additionally documented and reported using the SAE Report provided at the Initiation visit by the Sponsor or designee, as described in [(see Section 7.9.1.11)](#_Procedure_for_Reporting).

It is important that each SAE Report include a description of the event, its duration (onset and resolution dates/times), its severity, its relationship with the Medicinal Product, any other potential causal factors, any treatment given or other action taken (including dose modification or discontinuation of the Medicinal Product and its outcome. In addition, serious cases should be identified and the appropriate seriousness criteria documented.

As the quality and precision of acquired AE data are critical, Investigators should use the AE definitions provided in the above sections and should observe the following guidelines when completing the AE pages of the e-CRF:

- If a condition is a medical diagnosis, appropriate terminology should be used (e.g., high blood pressure should be reported as hypertension when a confirmed diagnosis exists)
- Any condition which contains more than one unique concept should be split into two or more events, or the condition should be described as a single medical diagnosis or concept (e.g., nausea and vomiting should be listed as two separate entries or as “gastroenteritis” when it is diagnosed as such)
- If a diagnosis is not available then the signs and symptoms should be recorded (e.g. if runny nose and itchy eyes have not been previously diagnosed as hay fever, then runny nose and itchy eyes should be recorded as two separate events)
- Any condition that could have various locations or types should be specified. Examples include:

Pain – left shoulder pain

Ache – generalized body aches or lower backache

Rash – macular rash on lower abdomen

Infection – lower left abdominal injection site infection or, upper respiratory infection; specify genus/species name of infecting organism if known (e.g., E.coli)

- If a subject has a worsening or exacerbation of a pre-existing condition previously reported in the medical history, include the appropriate terminology (i.e., worsening, exacerbation etc.)
- Avoid abbreviations as many abbreviations have more than one interpretation
- Any condition, which is ambiguous, must include additional information. Examples include:

Aches in hands – provide more specificity (i.e., muscle aches or joint aches or general)

Allergy – provide type (i.e., seasonal allergy or allergy to cats)

Angina – provide more specificity (i.e., angina pectoris or angina tonsilaris)

Chest pain – provide more specificity (i.e., musculoskeletal or cardiac or heartburn)

#### Recording Injections Site Reactions in the e-CRF

The most common AEs that may occur at the injection site are the following:

- Injection site redness
- Injection site pain
- Injection site bruising
- Injection site swelling
- Injection site itching
- More rarely, infection, abscess, cellulitis, necrosis

In order to ensure consistency in the reporting of these Injection Site Reactions (ISRs), the investigator is encouraged to use the above terminology, or specify any other type of ISR that the may observe in the course of treatment.

In case ISRs occur after every injection, they should be documented as one event which occurs intermittently during the trial, not as separate events.

#### Recording Flu-Like Symptoms in the e-CRF

The occurrence of flu-like symptoms (FLS) is recognized as a typical reaction to IFN- beta. FLS may include headache, fatigue, fever, chills, myalgia and arthralgia; typically, several of these symptoms would occur concomitantly. If investigators consider the symptoms experienced by the patient as being a FLS, they are encouraged to report the syndrome FLS rather than each individual symptom. If they occur individually, then they should not be reported as FLS. It is important to distinguish between FLS secondary to IFN-beta administration and true symptoms of flu, a viral infection, which would usually not abate within twenty-four hours.

#### Reporting of MS Relapses

In this trial, symptoms of MS relapses or worsening of MS will be recorded in the appropriate section of the e-CRF.

### Pregnancy and In Utero Drug Exposure

Only pregnancies considered by the Investigator as related to study treatment (e.g., resulting from a drug interaction with a contraceptive medication) are considered AEs. However, all pregnancies with an estimated conception date during the period defined in [Section 7.9.1.5](#_Definition_of_Serious) be recorded in the AE section of the e-CRF. The Investigator must notify the Sponsor in an expedited manner of any pregnancy using the Pregnancy Report Form (Part I), which must be transmitted according the same process as described for SAE reporting in [Section 7.9.1.11](#_Procedure_for_Reporting)*.*

Investigators must actively follow up, document and report on the outcome of all these pregnancies, even if the subjects are withdrawn from the trial. The Investigator must notify the Sponsor of these outcomes using the Pregnancy Report Form (Part II), and also must complete the SAE Report when the subject sustains an event and the Parent-Child Fetus Report Form when the child/fetus sustains an event.

Any abnormal outcome must be reported in an expedited manner as described in [Section 7.9.1.11](#_Procedure_for_Reporting), while normal outcomes must be reported within forty-five days from the date of delivery.

# Statistics

## Sample Size

The sample size is based on clinical rather than statistical considerations.

## Randomization

This study is not randomized; there is only one treatment group for RRMS patients.

## Analysis Sets

**Intention-to-treat (ITT)**

The ITT Population is the primary analysis population. All RRMS subjects with at least one injection of Rebif® and all HC who have signed the ICF will be included in the ITT Population.

**Per protocol**

The per protocol population includes all subjects who have been treated according to the clinical trial protocol and fulfill the following criteria:

- Compliance with all entry criteria
- Absence of major clinical trial protocol violations with respect to factors likely to affect the efficacy of treatment
- Adequate compliance with trial medication.

**Safety**

The safety population includes all ITT subjects.

## Description of Statistical Analyses

### General Considerations

All statistical tests will be two-tailed and considered significant at an α=0.05 level of significance; all confidence intervals will be two-sided 95% confidence intervals.

Dropouts, if any, will be examined. Subjects who stop the Medicinal Product will be asked to remain in the study and will be analyzed with the intention to treat, but adding a covariate that alternative therapies have been given. Individual data will be analyzed. No imputation of missing data will be performed.

T-tests and chi-square tests for independent samples will be used for comparison of demographic and clinical characteristics between RRMS patients and HC.

VW-MTR and SWI spatial distribution analysis will be conducted directly on the images. Analysis of MRI measures may require a variance stabilizing transformation, if skewness is observed in their assessments. Each of the MRI parameters will be examined and appropriate transformations will be applied, if necessary.

### Analysis of Primary Endpoint(s)

The primary endpoint, the per-voxel remyelination/demyelination count changes in NABT at 12 and 24 weeks will be summarized for each group with descriptive statistics: mean, standard deviation, median, minimum and maximum and a two-sided 95% confidence interval. For testing differences in remyelination/demyelination between groups, an analysis of covariance (ANCOVA) on the ranked data with effects for treatment group (RRMS vs. HC), treatment period (1^st^ 3 months vs. 2^nd^ 3 months), age, and gender will be performed. Model assumptions of linearity, normality and homoschedasticity will be verified. For testing differences across time (at week 12 and 24) within the RRMS treatment group, the Wilcoxon signed rank test will be used.

Exploratory analyses on the primary endpoint may be performed to include other covariates of interest, such as time since MS diagnosis, immune status, etc.

### Analysis of Secondary Endpoint(s)

Secondary endpoints will be summarized at Baseline (if available), Week 12 and Week 24/Exit, using the same descriptive statistics used to analyze the primary endpoint as described above. Testing differences between groups and across time will be analyzed similarly to the primary endpoint.

Further details will be provided in a separate Statistical Analysis Plan (SAP), which will be finalized prior to performing any analyses.

### Safety Analyses

Safety will be assessed through the evaluation of endpoints related to adverse events, laboratory assessments, thyroid function tests, and vital signs.

Adverse event frequencies and numbers of subjects with adverse events will be summarized by system organ class and preferred term. Additional summaries will be presented by severity as well as by relationship to the Medicinal Product. Laboratory results will be summarized by incidence and toxicity grade and by change from Baseline to Week 24/Exit. Vital signs will be summarized descriptively.

### Analysis of Further Endpoints

Not applicable.

## Interim Analysis

Not applicable.

# Ethical and Regulatory Aspects

## Responsibilities of the Investigator

The Investigator is responsible for the conduct of the trial at his/her site. He/she will ensure that the trial is performed in accordance with the clinical trial protocol and with the ethical principles that have their origin in the Declaration of Helsinki, as well as with the ICH Guideline on Good Clinical Practice (ICH Topic E6, 1996) and applicable regulatory requirements. In particular, the Investigator must ensure that only subjects who have given their informed consent are included into the trial.

In 1998, the US Food and Drug Administration (FDA) introduced a regulation (21 CFR, Part 54) entitled “Financial Disclosure by Clinical Investigators”. For trials conducted in any country that could result in a product submission to the FDA for marketing approval and could contribute significantly to the demonstration of efficacy and safety of the drug (named “covered trials” by the FDA), the Investigator and all sub-Investigators are obliged to disclose any financial interest which they, their spouses or their dependent children may have in the Sponsor or the Sponsor’s product under study. This information is required during the trial and for twelve months following completion of the trial.

## Subject Information and Informed Consent

An unconditional prerequisite for a subject’s participation in the trial is his/her written informed consent. The subject’s written informed consent to participate in the trial must be given before any trial-related activities are carried out.

Adequate information must therefore be given to the subject by the Investigator before informed consent is obtained (a person designated by the Investigator may give the information, if permitted by local regulations). A subject information sheet in the local language and prepared in accordance with the Note for Guidance on Good Clinical Practice (ICH Topic E6, 1996) will be provided by the Sponsor for the purpose of obtaining informed consent. In addition to providing this written information to a potential subject, the Investigator or his/her designate will inform the subject verbally of all pertinent aspects of the trial. The language used in doing so must be chosen so that the information can be fully and readily understood by lay persons.

Depending on national regulations, a person other than the Investigator may inform the subject and sign the Informed Consent Form (ICF), as above.

Where the information is provided by the Investigator, the ICF must be signed and personally dated by the subject and the Investigator.

The signed and dated declaration of informed consent will remain at the Investigator’s site, and must be safely archived by the Investigator so that the form can be retrieved at any time for monitoring, auditing and inspection purposes. A copy of the signed and dated information and ICF should be provided to the subject prior to participation.

Whenever important new information becomes available that may be relevant to the subject’s consent, the written subject information sheet and any other written information provided to subjects will be revised by the Sponsor and be submitted again to the IRB for review and favorable opinion. The agreed, revised information will be provided to each subject in the trial for signing and dating. The Investigator will explain the changes to the previous version.

## Subject Identification and Privacy

A unique subject number will be assigned to each subject at inclusion, immediately after informed consent has been obtained. This number will serve as the subject’s identifier in the trial as well as in the clinical trial database.

The subject’s data collected in the trial will be stored under this number. Only the Investigator will be able to link the subject’s trial data to the subject via an identification list kept at the site. The subject’s original medical data that are reviewed at the site during source data verification by the Monitor, audits and Health Authority inspections will be kept strictly confidential.

Data protection and privacy regulations will be observed in capturing, forwarding, processing, and storing subject data. Subjects will be informed accordingly, and will be requested to give their consent on data handling procedures in accordance with national regulations.

## Emergency Medical Support and Subject Card

Subjects enrolled in this clinical trial will be provided with Emergency Medical Support cards during their trial participation, which will be furnished by the Sponsor. The Emergency Medical Support card is based on the need to provide clinical trial subjects with a way of identifying themselves as participating in a clinical trial, and subsequently to give health care providers access to the information about this participation that may be needed to determine the course of the subject’s medical treatment.

This service is designed to provide information to health care providers who are not part of the clinical trial. Clinical trial Investigators, who are already aware of the clinical trial protocol and treatment, have other means of accessing the necessary medical information for the management of emergencies occurring in their subjects.

The first point of contact for all emergencies will be the clinical trial Investigator caring for the affected subject. The Investigator agrees to provide his or her emergency contact information on the card for this purpose. If the Investigator is available when an event occurs, she will answer any questions. Any subsequent action will follow the standard processes established for the Investigators.

In cases where the Investigator is not available, Merck Serono/EMD Serono provides the appropriate means to contact a Sponsor physician. This includes the provision of a 24 hour contact number at a call centre, whereby the health care providers will be given access to the appropriate Sponsor physician to assist with the medical emergency and to provide support for the subject concerned.

## Institutional Review Board

Prior to commencement of the trial at a given site, the clinical trial protocol will be submitted together with its associated documents (e.g., Subject Information Sheet and Informed Consent Form (ICF), and any other requested documents*)* to the responsible Institutional Review Board (IRB) for its approval. The written approval of the IRB will be filed in the Investigator File and a copy will be filed in the Sponsor’s Trial Master File*.*

The trial must not start at a site before the Sponsor has obtained written confirmation of approval from the concerned IRB. The IRB will be asked to provide documentation of the date of the meeting at which the approval was given, and of the members and voting members present at the meeting. Written evidence of approval that clearly identifies the trial, the clinical trial protocol version and the Subject Information and ICF version reviewed should be provided. Where possible, copies of the meeting minutes should be obtained.

Amendments to the protocol will also be submitted to the concerned IRB, before implementation in case of substantial changes [(see Section 10.5)](#_Changes_to_the). Relevant safety information will be submitted to the IRB during the course of the trial in accordance with national regulations and requirements.

## Health Authorities

Not applicable

# Trial Management

## Case Report Form Handling

The Investigator or designee will be responsible for entering trial data in the electronic CRF (e-CRF) provided by the Sponsor. It is the Investigator’s responsibility to ensure the accuracy of the data entered in the e-CRFs.

The data will be entered into a validated database, and the Sponsor, or designee, will be responsible for data processing, in accordance with the Sponsor’s, or designee’s, data management procedures. The database lock will occur once quality assurance procedures have been completed. Portable Document Format (PDF) files of the e-CRFs will be provided to the Investigators at the completion of the trial.

## Source Data and Subject Files

The Investigator must keep a subject file (medical file, original medical records) on paper or electronically for every subject included in the trial. This file will contain the available demographic and medical information for the subject, and should be as complete as possible. In particular, the following data should be available in this file: (adapt to trial as necessary)

- Subject’s full name,
- Date of birth,
- Sex,
- Height,
- Weight,
- Medical history and concomitant diseases,
- Prior and concomitant therapies (including changes during the trial),
- Trial identification (Sponsor’s trial number according to clinical trial protocol),
- Date of subject’s inclusion into the trial (i.e. date of giving informed consent),
- Subject number in the trial,
- Subject number (specify as appropriate),
- Dates of the subject’s visits to the site,
- Any medical examinations and clinical findings predefined in the clinical trial protocol,
- All adverse events observed in the subject,
- Date of subject’s end of trial, and
- Date of and reason for early withdrawal of the subject from the trial or from drug, if applicable.

It must be possible to identify each subject by using this subject file.

Additionally, any other documents containing source data must be filed. This includes value listings of subjects’ results from the scheduled laboratory tests and MRI scan images. Such documents must bear at least the subject number and the date when the procedure was performed. Information should be printed by the instrument used to perform the assessment or measurement, if possible. Information that cannot be printed by an automated instrument will be entered manually. Medical evaluation of such records should be documented as necessary and the documentation signed and dated by the Investigator.

Electronic subject files will be printed whenever the Monitor performs source data verification. Printouts must be signed and dated by the Investigator, countersigned by the Monitor and kept in a safe place at the site.

### Hospitalization Records

Subjects may be hospitalized for any reason at the discretion of the Investigator. In case of

hospitalization, the following items will be documented in the Serious Adverse Event Report:

- Reason for hospitalization
- MS relatedness
- Date of admission
- Date of discharge

Each hospitalization constitutes a Serious Adverse Event [(see Section 7.9.1.8)](#_Definition_of_Serious)

## Investigator Site File and Archiving

The Investigator will be provided with an Investigator Site File upon initiation of the trial. This file will contain all documents necessary for the conduct of the trial and will be updated and completed throughout the trial. It must be available for review by the Monitor, and must be ready for Sponsor audit as well as for inspection by Health Authorities during and after the trial, and must be safely archived for at least 15 years (or per local requirements or as otherwise notified by the Sponsor) after the end of the trial. The documents to be thus archived include the Subject Identification List and the signed subject Informed Consent Forms. If archiving of the Investigator Site File is no longer possible at the site, the Investigator must notify the Sponsor.

All original subject files (medical records) must be stored at the site (hospital, research institute, or practice) for the longest possible time permitted by the applicable regulations, and/or as per ICH GCP guidelines, whichever is longer. In any case, the Investigator should ensure that no destruction of medical records is performed without the written approval of the Sponsor.

## Monitoring, Quality Assurance and Inspection by Health Authorities

This trial will be monitored in accordance with the ICH Guideline on Good Clinical Practice (ICH Topic E6, 1996). The site Monitor will perform visits to the trial site at regular intervals.

Representatives of the Sponsor’s Quality Assurance unit or a designated organization, as well as Health Authorities, must be permitted to inspect all trial-related documents and other materials at the site, including the Investigator Site File, the completed e-CRF, the drug(s), and the subjects’ original medical records/files.

The clinical trial protocol, each step of the data capture procedure, and the handling of the data, including the final clinical trial report, will be subject to independent Quality Assurance activities. Audits may be conducted at any time during or after the trial to ensure the validity and integrity of the trial data.

## Changes to the Clinical Trial Protocol

Changes to the clinical trial protocol will be documented in written protocol amendments. Major (substantial, significant) amendments will usually require submission to the relevant IRB for approval or favorable opinion. In such cases, the amendment will be implemented only after approval or favorable opinion has been obtained.

Minor (nonsubstantial) protocol amendments, including administrative changes, will be filed by the Sponsor and at the site. They will be submitted to the relevant IRB or to Health Authorities only where required by pertinent regulations.

Any amendment that could have an impact on the subject’s agreement to participate in the trial requires the subject’s informed consent prior to implementation [(see Section 9.2)](#_Subject_Information_and)

## Clinical Trial Report and Publication Policy

### Clinical Trial Report

After completion of the trial, a clinical trial report according to ICH Topic E3 Structure and Content of Clinical Study Reports will be written by the Sponsor or designate in consultation with the Coordinating Investigator/the Principal Investigator.

### Publication

The first publication will be a publication of the results of the analysis of the primary endpoint(s) that will include data from the trial site.

The Investigator will inform the Sponsor in advance about any plans to publish or present data from the trial. Any publications and presentations of the results (abstracts in journals or newspapers, oral presentations, etc.), either in whole or in part, by Investigators or their representatives will require pre-submission review by the Sponsor.

The Sponsor will not suppress or veto publications, but maintains the right to delay publication in order to protect intellectual property rights.

# References

(1) De Stefano N, Narayanan S, Francis SJ, Smith S, Mortilla M, Tartaglia MC et al. Diffuse axonal and tissue injury in patients with multiple sclerosis with low cerebral lesion load and no disability. Arch Neurol 2002; 59:1565-1571.

(2) Barkhof F, Bruck W, De Groot CJ, Bergers E, Hulshof S, Guerts J et al. Remyelinated lesions in multiple sclerosis: magnetic resonance image appearance. Arch Neurol 2003; 60:1073-1081.

(3) Zivadinov R. Can imaging techniques measure neuroprotection and remyelination in mutliple sclerosis. Neurology 2007; 68:S72-S82.

(4) Dutta R, Trapp BD. Pathogenesis of axonal and neuronal damage in multiple sclerosis. Neurology 2007; 68:S22-S31.

(5) Tjoa CW, Benedict RH, Dwyer MG, Carone DA, Zivadinov R. Regional specificity of magnetization transfer imaging in multiple sclerosis. J Neuroimaging 2008; 18(2):130-136.

(6) Chen JT, Collins DL, Atkins HL, Freedman MS, Arnold DL, Canadian MS/BMT Study Group. Magnetization transfer ratio evolution with demyelination and remyelination in multiple sclerosis lesions. Ann Neurol 2008; 63:254-262.

(7) Patrikos P, Stadelmann C, Kutzelnigg A, Rauschka H, Schmidbauer M, Lursen H et al. Remyelination is extensive in a subset of multiple sclerosis patients. Brain 2006; 129:3165-3172.

(8) Schmierer K, Scaravilli F, Altmann DR, Barker GJ, Miller DH. Magnetization transfer ratio and myelin in postmortem multiple sclerosis brain. Ann Neurol 2004; 56:407-415.

(9) Deloire-Grassin MSA, Brochet B, Quesson B, Delalande C, Dousset V, et al. In vivo evaluation of remyelination in rat brain by magnetization transfer imaging. J Neuro Sci 2000; 178:10-16.

(10) Audoin B, Davies G, Rashid W, Fisniku L, Thompson AJ, Miller DH. Voxel-based analysis of grey matter magnetization transfer ratio maps in early relapsing remitting multiple sclerosis. Mult Scler 2007; 13:483-489.

(11) Chen JT, Kuhlmann T, Jansen GH, Collins DL, Atkins HL, Freedman MS et al. Voxel-based analysis of the evolution of magnetization transfer ratio to quantify remyelination and demyelination with histopathological validation in a multiple sclerosis lesion. Neuroimage 2007; 36:1152-1158.

(12) Zaaraoui W, Deloire M, Merle M, Girard C, Raffard G, Biran M et al. Monitoring demyelination and remyelination by magnetization transfer imaging in the mouse brain at 9.4 T. Magn Reson Mater Phy 2008; 21:357-362.

(13) DeStefano N, Battaglini M, Stromillo ML, Zipoli V, Bartolozzi ML, Guidi L. Brain damage as detected by magnetization transfer imaging is less pronounced in benign than in early relapsing multiple sclerosis. Brain 2006; 129:2008-2016.

(14) Filippi M, Rocca MA, Martino G, Horsfield MA, Comi G. Magnetization transfer changes in the normal appearing white matter precede the appearance of enhancing lesions in patients with multiple sclerosis. Ann Neurol 1998; 43:809-814.

(15) Lin X, Tench CR, Morgan PlS, Constantinescu CS. Use of combined conventional and quantitative MRI to quantify pathology related to cognitive impairment in multiple sclerosis. J Neurol Neurosurg Psychiatry 2008; 79:437-441.

(16) Melzi L, Rocca MA, Marzoli SB, Falini A, Vezzulli P, Ghezzi A et al. A longitudinal conventional and magnetization transfer magnetic resonance imaging study of optic neuritis. Mult Scler 2007; 13:265-268.

(17) Mottershead JP, Schmierer K, Clemence M, thornton JS, Scaravilli F, et al. High field MRI correlates of myelin content and axonal density in multiple sclerosis: a post-mortem study of the spinal cord. J Neurol 2003; 250:1293-1301.

(18) Rovaris M, Agosta F, Sormani MP, Inglese M, Martinelli V, Comi G et al. Conventional and magnetization transfer MRI predictors of clinical multiple sclerosis evolution: a medium-term follow-up study. Brain 2003; 126:2323-2332.

(19) Santos AC, Narayanan S, De Stefano N, Tartaglia MC, Francis SJ, et al. Magnetization transfer can predict clinical evolution in patients with multiple sclerosis. J Neurol 2002; 249:662-668.

(20) Sharma J, Zivadinov R, Jaisani Z, Fabiano AJ, Siingh B, et al. A magnetization transfer MRI study of deep gray matter involvement in multiple sclerosis. J Neuroimaging 2006; 16(4):302-310.

(21) Zivadinov R, de Masi R, Nasuelli D, Bragadin LM, Ukmar M, Pozzi-Mucelli RS et al. MRI techniques and cognitive impairment in the early phase of relapsing-remitting multiple sclerosis. Neuroradiology 2001; 43:272-278.

(22) Ashburner J, Friston KJ. Voxel-based morphometry--the methods. Neuroimage 2000; 11:805-821.

(23) Dwyer M, Bergsland N, Hussein S, Durfee J, Wack D, Zivadinov R. A sensitive,noise-resistant method for identifying focal demyelination and remyelination in patients with multiple sclerosis via voxel-wise changes in magnetization transfer ratio. J Neuro Sci 2009; 282(86):95.

(24) Bink A, Schmitt M, Gaa J, Mugler JP3, Lanfermann H, Zanella FE. Detection of lesions in multiple sclerosis by 2D FLAIR and single-slab 3D FLAIR sequences at 3.0 T: initial results. Eur Radiol 2006; 16:1104-1110.

(25) Guerts JJ, Pouwels PJ, Uitedehaag BM, Polman CH, Barkhof F, Castelijns JA. Intracortical lesions in multiple sclerosis: improved detection with 3D double inversion-recovery MR imaging. Radiology 2005; 236:254-260.

(26) Vellinga MM, Engberink RD, Seewann A, Pouweis PJ, Wattjes MP, van der Pol SM et al. Pluriformity of inflammation in multiple sclerosis shown by ultra-small iron oxide particle enhancement. Brain 2008; 131:800-807.

(27) Guerts JJ, Barkhof F. Grey matter pathology in multiple sclerosis. Lancet Neurol 2008; 7:841-851.

(28) Kutzelnigg A, Luccinetti CF, Stadelmann C, Bruck W, Rauschka H, Bergmann M et al. Cortical demyelination and diffuse white matter injury in multiple sclerosis. Brain 2005; 128:2705-2712.

(29) Chen JT, Collins DL, Atkins HL, Freedman MS, Arnold DL. Detection of subpial cortical demyelinating lesions in multiple sclerosis in vivo. Proc Intl Soc Mag Reson Med 2006; 14:2094.

(30) Haacke EM, Makki M, Ge Y, Maheshwari M, Sehgal V, Hu J et al. Characterizing iron deposition in multiple sclerosis lesions using susceptibility weighted imaging. J Magn Reson Imaging 2009; 29(3):537-544.

(31) Thomas B, Somasundaram S, Thamburaj K, Kesavadas C, Gupta AK, Bodhey NK et al. Clinical applications of susceptibility weighted MR imaging of the brain-a pictorial review. Neuroradiology 2008; 50:105-116.

(32) Ge Y, Zohrabian VM, Grossman RI. Seven-telsa magnetic resonance imaging: new vision of microvascular abnormalities in multiple sclerosis. Arch Neurol 2008; 65(6):812-816.

(33) Hammond KE, Lupo JM, Xu D, Metcalf M, Kelley DAC, Pelletier D et al. Development of a robust method for generating 7.0 T multichannel phase images of the brain with application to normal volunteers and patients with neurological diseases. Neuroimage 2008; 39:1682-1692.

(34) PRISMS Study Group. Randomised double-blind placebo-controlled study of interferon b-1a in relapsing/remitting multiple sclerosis. Lancet 1998; 352(9139):1498-1504.

(35) PRISMS Study Group, University of British Columbia MS/MRI Analysis Group. PRISMS-4: Long-term efficacy of interferon-b-1a in relapsing MS. Neurology 2001; 56(12):1628-1636.

(36) Sullivan JL. Is stored iron safe? J Lab Clin Med 2004; 144:280-284.

(37) Giaume C, Kirchhoff F, Matute C, Reichenbach A, Verkhratsky A. Glia: the fulcrum of brain diseases. Cell Death Differ 2007; 14:1324-1335.

(38) Paty DW, Ebers GC. Clinical Features. In: Paty DW, Ebers GC, editors. Multiple Sclerosis. Philadelphia: I.A.Davis Company, 1997: 135-191.

(39) Acheson ED. Epidemiology of multiple sclerosis. Br Med Bull 1977; 33(1):9-14.

(40) Phadke JG, Downie AW. Epidemiology of multiple sclerosis in the north-east (Grampian region) of Scotland - an update. J Epidemiol Community Health 1987; 41(1):5-13.

(41) Phadke JG, Downie AW. Epidemiology of multiple sclerosis in the north-east (Grampain Region) of Scotland--an update. J Epidemiol Comm Health 1987; 41:5-13.

(42) Ebers GC, Sadovnick AD. The role of genetic factors in multiple sclerosis susceptibility. J Neuroimmunol 1994; 54(1):1-17.

(43) Sadovnick AD, Ebers GC, Dyment DA, Risch NJ, Canadian Collaborative Study Group. Evidence for genetic basis of multiple sclerosis. Lancet 1996; 347:1728-1730.

(44) Sadovnick AD, Ebers GC. Epidemiology of multiple sclerosis: A critical overview. Can J Neurol Sci 1993; 20(1):17-29.

(45) Ota K, Matsui M, Milford EL, Mackin GA, Weiner HL, Hafler DA. T-cell recognition of an immunodominant myelin basic protein epitope in multiple sclerosis. Nature 1990; 346:183-187.

(46) Martin R, McFarland HF, McFarlin DE. Immunological aspects of demyelinating diseases. Annu Rev Immunol 1992; 10:153-187.

(47) Beck J, Rondot P, Catinot L, Falcoff E, Kirchner H, Wietzerbin J. Increased production of interferon-gamma and tumour necrosis factor precedes clinical manifestation in multiple sclerosis: Do cytokines trigger off exacerbations? Acta Neurol Scand 1988; 78:318-323.

(48) Lublin FD, Reingold SC. Defining the clinical course of multiple sclerosis: Results of an international survey. Neurology 1996; 46:907-911.

(49) Runmarker B, Andersen O. Prognostic factors in a multiple sclerosis incidence cohort with twenty-five years of follow-up. Brain 1993; 116:117-134.

(50) Li DKB, Paty DW. Magnetic resonance imaging results of the PRISMS trial: a randomized, double-blind, placebo-controlled study of interferon-b1a in relapsing-remitting multiple sclerosis. Neurology 1999; 46:197-206.

(51) Li DKB, Paty DW, PRISMS Study Group. Magnetic resonance imaging results of the PRISMS trial: A randomized, double-blind, placebo-controlled study of interferon-b1a in relapsing-remitting multiple sclerosis. Ann Neurol 1999; 46(2):197-206.

(52) Panitch H, Goodin DS, Francis G, Chang P, Coyle PK, O'Connor P. Randomized, comparative study of interferon b-1a treatment regimens in MS. The EVIDENCE trial. Neurology 2002; 59(10):1496-1506.

(53) Zivadinov R, Sepcic J, Nasuelli D, de Masi R, Bragadin LM, et al. A longitudinal study of brain atrophy and cognitive disturbances in the early phase of relapsing-remitting multiple sclerosis. J Neurol Neurosurg Psychiatry 2001; 70:773-780.

(54) Barkhof F, Filippi M, Van Waesberghe JH, Molyneux P, Rovaris M, Lycklama et al. Improving interobserver variation in reporting gadolinium-enhanced MRI lesions in multiple sclerosis. Neurology 1997; 49:1682-1688.

(55) Zivadinov R, Bagnato F, Nasuelli D, Bastianello S, Bratina A, Locatelli L et al. Short-term brain atrophy changes in relapsing-remitting multiple sclerosis. J Neurol Sci 2004; 223:185-193.

(56) Smith SM, Zhang Y, Jenkinson M, Chen J, Matthews PM, Federico A et al. Accurate, robust, and automated longitudinal and cross-sectional brain change analysis. Neuroimage 2002; 17:479-489.

(57) Frexes-Steed M, Lacy DB, Collins J, Abumrad NN. Role of leucine and other amino acids in regulating protein metabolism in vivo. Am J Physiol 1992; 262:E925-E935.

(58) Zhang Y, Brady M, Smith S. Segmentation of brain MR images through a hidden Markov random field model and the expectation-maximization algorithm. IEEE Trans Med Imaging 2001; 20(1):45-57.

# Appendices

## Appendix A - Schedule of Assessments

| RRMS Subjects | | | | | |
| --- | --- | --- | --- | --- | --- |
| **Study Phase** | **Screening** | **Baseline** | **Treatment** | | **Telephone** |
| Visit | 1 | 2 | 3 | 4 | 5 |
| **Study Procedure** | **within 14 days** | **Study**  **Day 1** | **Week 12 (3 Mos.)** | **Week 24/Exit (6 Mos.)** | **Safety Follow-up Call** |
| Informed Consent/HIPAA/ Eligibility Criteria | X |  |  |  |  |
| Medical History/Demographics | X |  |  |  |  |
| Physical/ Neuro Exam | X^1^ |  | X | X |  |
| Laboratory Assessments (chem. heme, ferritin, uric acid) | X |  | X | X |  |
| Thyroid Function Tests^2^ | X |  |  | X |  |
| Adverse Events/ Concomitant Meds/Procedures | X | X | X | X | X |
| Pregnancy Testing (Serum) | X |  |  |  |  |
| Immunological Samples |  | X |  | X |  |
| Samples Shipment |  | X |  | X |  |
| Pregnancy Testing (Urine) |  | X |  | X |  |
| Drug Dispensing/Accountability |  | X | X | X |  |
| Injection Training |  | X |  |  |  |
| MRI |  | X | X | X |  |

1 Includes EDSS (screening visit only)

2 Thyroperoxidase Antibody test only analyzed if T3, T4 or TSH were elevated

| Healthy Controls | | | | | |
| --- | --- | --- | --- | --- | --- |
| **Study Phase** | **Screening** | **Baseline** | **Treatment** | | **Telephone** |
| Visit | 1 | 2 | 3 | 4 | 5 |
| **Study Procedure** | **within 14 days** | **Study**  **Day 1** | **Week 12 (3 Mos.)** | **Week 24/Exit (6 Mos.)** | **Safety Follow-up Call** |
| Informed Consent/HIPAA/ Eligibility Criteria | X |  |  |  |  |
| Medical History/Demographics | X |  |  |  |  |
| Adverse Events/ Concomitant Meds/Procedures | X | X | X | X | X |
| Physical/Neuro Exam | X |  | X | X |  |
| Laboratory Assessments (chem. heme, ferritin, serum pregnancy) | X |  |  |  |  |
| MRI |  | X | X | X |  |
| Immunological Samples |  | X |  |  |  |
| Pregnancy Testing (Urine) |  | X |  | X |  |
| Samples Shipment |  | X |  |  |  |

## Appendix B - Laboratory Parameters

| Hematology- CBC w/diff | **Blood Chemistry** | **Special Chemistries** |
| --- | --- | --- |
| White Blood Cell Count | Albumin | Ferritin |
| Red Blood Cell Count | AST | Transferrin |
| Hemoglobin | ALT | Uric Acid |
| Hematocrit | Alkaline Phosphatase | Immunological Samples |
| Platelet Count | Amylase | Th17 pathway-related cytokines; growth factors |
| Percent Segmented Neutrophils | Bilirubin Direct & Total |  |
| Band Neutrophils | Blood Urea Nitrogen |  |
|  | Calcium |  |
| **Urinalysis** | Carbon Dioxide |  |
| Color | Chloride |  |
| Clarity | Creatine Serum |  |
| Specific Gravity | Glucose |  |
| pH | Iron |  |
| Glucose | Potassium |  |
| Ketones | Sodium | Hormonal Panel |
| Protein | Total Protein | Anti-Thyroid Peroxidase Antibody^1^ |
| Bilirubin |  | Triiodothyronine (T3) |
| Occult Blood | Lipase | Thyroxine (T4) |
| Leukocytes | **Pregnancy Testing** | Thyroid Stimulating Hormone (TSH) |
| Nitrites | hCG |  |
| Microscopic Analysis | Urine |  |

1- to be done if T_3_, T_4_ or TSH are elevated

## Appendix C- Common Terminology Criteria for Adverse Events

The Common Terminology Criteria for Adverse Events (CTCAE) v3.0 will be provided in a separate document.
